# Design Rules for Quantitative Control of Intragrain Planar Defects in Halide Perovskites

**DOI:** 10.1002/advs.77365

**Published:** 2026-08-24

**Authors:** Byeongjun Gil, So Jeong Park, Jin Young Kim, Miyoung Kim

**Affiliations:** ^1^ Department of Materials Science and Engineering Seoul National University Seoul Republic of Korea; ^2^ Research Institute of Advanced Materials (RIAM) Seoul National University Seoul Republic of Korea; ^3^ School of Transdisciplinary Innovations Seoul National University Seoul Republic of Korea

**Keywords:** defect engineering, halide perovskites, intragrain planar defects, low‐dose transmission electron microscopy, perovskite solar cells

## Abstract

Intragrain planar defects limit carrier transport and operational stability in halide perovskite photovoltaics, yet quantitative rules to control their formation are still lacking. Here defect‐specific design rules are established for two dominant planar defects—{112}_t_ ferroelastic twins and {111}_c_ twins/stacking faults—in MA_1‐x_Gua_x_PbI_3_ thin films (MA = methylammonium, Gua = guanidinium) by rigorously dose‐budgeted low‐dose transmission electron microscopy. Independent variation of A‐site composition and annealing conditions decouples the roles of tetragonality (*t*; pseudo‐cubic *c*/*a*), grain size, and crystallization kinetics. {112}_t_ twin density increases with tetragonality and grain size but is insensitive to crystallization kinetics, whereas {111}_c_ defects emerge at reduced tetragonality and remain grain‐size independent. These correlations define a processing window (*t* ≈ 1.005–1.010 for submicron grains) that simultaneously suppresses both defect types. Solar cells fabricated within this window exhibit longer carrier lifetimes and higher power‐conversion efficiencies, supporting the importance of intragrain planar‐defect control.

## Graphical abstract

Dose‐budgeted electron microscopy reveals the design rules governing intragrain planar defects in halide perovskites: {112}_t_ ferroelastic twins proliferate with increasing tetragonality and grain size, whereas {111}_c_ twins and stacking faults emerge as the lattice approaches cubic symmetry. Between these regimes lies a defect‐free window where carrier lifetimes lengthen and power‐conversion efficiency rises.

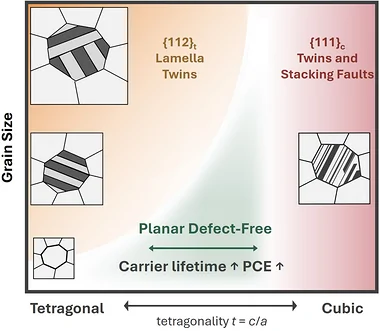

## 1 Introduction

Metal halide perovskites combine outstanding optoelectronic performance with solution‐processability, making them promising candidates for next‐generation photovoltaics [1, 2]. Although the solution processing inherently yields high defect densities [3, 4], theoretical studies suggest that most bulk point defects form shallow trap states, indicating intrinsic defect tolerance [5, 6, 7]. However, defects at surfaces, interfaces, and grain boundaries introduce altered bonding configurations that create deep trap states, exacerbate non‐radiative recombination, and ultimately degrade device performance [4, 8, 9, 10, 11, 12]. Accordingly, extensive compositional and additive engineering has focused on passivating these interfacial defects, achieving substantial gains in carrier mobility and power‐conversion efficiency [13, 14, 15, 16, 17, 18, 19].

Defects within bulk grains, such as twins and stacking faults, can also induce local structural distortions and modified bonding character. They interact with nearby point defects and critically influence carrier dynamics [20, 21, 22, 23, 24]. The most prevalent examples are the {111}_c_‐type twins and stacking faults in cubic phases (e.g. α‐FAPbI_3_; FA denotes formamidinium) and {112}_t_‐type ferroelastic twins in tetragonal or orthorhombic phases (e.g. MAPbI_3_ or CsPbBr_3_, where MA denotes methylammonium). The {111}_c_‐types have been shown to impair carrier transport [25, 26, 27], through intrinsic hole‐blocking behavior [28, 29], or defect accumulation at boundaries [30, 31, 32]. Although {112}_t_‐type defects are often regarded as less detrimental [33] due to their small twin angle (∼1°) [34], they still hinder carrier diffusion across the twin wall [35], indicating active trapping behavior. The detrimental impacts of both defect types on photovoltaic performance have been demonstrated in FA_x_MA_1‐x_PbI_3_ films [21], challenging the claims of beneficial ferroelasticity‐related effects [36] or presumed ferroelectric contributions [37, 38].

Strategies to mitigate planar defects have primarily relied on compositional optimization and stringent control of crystallization. A‐site cation mixing suppresses {111}_c_‐ and/or {112}_t_‐type defects in Cs_x_FA_1‐x_PbI_3_ [25], and FA_x_MA_1‐x_PbI_3_ [21], while ionic liquid additives [39], antisolvent additives [27], and vapor treatments [21, 40], suppress {111}_c_‐type defects by modulating nucleation and growth. However, establishing defect‐specific formation rules requires statistically meaningful measurements of defect density, type, and morphology across a wide range of compositions and annealing protocols—a task hindered by the intrinsic beam sensitivity of halide perovskites [24, 41, 42, 43, 44, 45, 46, 47, 48, 49]. Conventional TEM imaging can readily induce, transform, or erase planar defects, particularly {112}_t_ twins, at cumulative doses of only a few e^−^ Å^−^^2^ [50, 51], so an apparently “defect‐free” grain cannot be unambiguously interpreted: it may be genuinely twin‐free or may have lost its twins during imaging. Overcoming this requires defect‐specific electron‐beam damage thresholds and imaging under low‐dose conditions tailored to each defect type, an experimental complexity that has thus far left most defect‐engineering strategies empirical in nature.

Here, we address this gap using low‐dose transmission electron microscopy (TEM), with beam damage thresholds determined separately for each defect type, to establish quantitative correlations between processing parameters and planar defect densities in MA_1‐x_Gua_x_PbI_3_ (Gua = guanidinium) films. Low‐dose 4D‐STEM nanobeam diffraction, in particular, provides a grain‐by‐grain statistical measure of {111}_c_ defects, which are otherwise difficult to count in bright‐field imaging. Through independent variation of A‐site composition (0%–25% Gua) and annealing conditions, we decouple the effects of tetragonality (*t*; expressed as pseudo‐cubic *c*/*a*), grain size, and crystallization kinetics. We show that the {112}_t_ twin density is governed primarily by grain size and tetragonality, consistent with thermodynamic domain scaling, and remains insensitive to crystallization kinetics. By contrast, {111}_c_ defects are promoted by reduced tetragonality and appear to be further favored by rapid crystallization, yet remain independent of grain size. These insights define a processing window that simultaneously suppresses both defect types. Photovoltaic devices fabricated under these conditions (10%–15% Gua, IA30 annealing) exhibit prolonged carrier lifetimes and enhanced power conversion efficiencies. Together, these results establish actionable design rules—modulating tetragonality and crystallization dynamics—for minimizing intragrain planar defects and improving halide perovskite photovoltaic efficiency.

## 2 Results and Discussion

Because intragrain planar defects in halide perovskites are readily modified by electron irradiation, we first calibrated defect‐specific beam‐damage thresholds and implemented strict dose budgeting for all TEM measurements (Note S1; Figures S1 and S2). In MAPbI_3_, the perovskite lattice remains largely stable below ∼10 e^−^ Å^−^^2^ (Figure S1) [44, 49], yet {112}_t_ ferroelastic domains begin to deform at ∼3.5 e^−^ Å^−^^2^ and nearly disappear by ∼5.9 e^−^ Å^−^^2^ (Figure S2a). We therefore acquired each BF‐TEM image from a previously unexposed region with a total dose ≤ 1 e^−^ Å^−^^2^, enabling statistically meaningful quantification of twinning probability and lamella geometry over a large grain population. In contrast, {111}_c_ planar defects tolerate substantially higher doses (Figure S2b), allowing complementary atomic‐resolution verification under ultralow‐dose HAADF‐STEM. This calibrated workflow underpins all defect‐density trends and the processing window established below.

### 2.1 Formation Parameters of {112}_t_‐type Planar Defects

The {112}_t_ twins in tetragonal halide perovskites correspond to ferroelastic domain walls formed to accommodate the tetragonal distortion [34]. The factors that determine domain structures govern the dominant twin boundary orientation and density, which are directly linked to charge‐carrier transport and, in turn, photovoltaic performance. In ferroic systems such as ferroelectrics and ferromagnetics, domain structures are generally determined by grain or crystallite size [52]. To examine the size dependence of {112}_t_ ferroelastic twin domains, we directly visualized domains in tetragonal MAPbI_3_ films using low‐dose bright‐field (BF) TEM. All BF‐TEM images were acquired using the calibrated low‐dose protocol described above (total dose ≤ 1 e^−^ Å^−^^2^; Note S1 and Figures S1 and S2). Figure 1a–d show BF‐TEM images of MAPbI_3_ films spin‐coated onto carbon‐film TEM grids (see Methods for preparation details). The observed lamellar stripes were identified as ferroelastic domains bounded by {112}_t_ twin planes [34], as confirmed by diffraction analysis (Figure S3).

**FIGURE 1:**
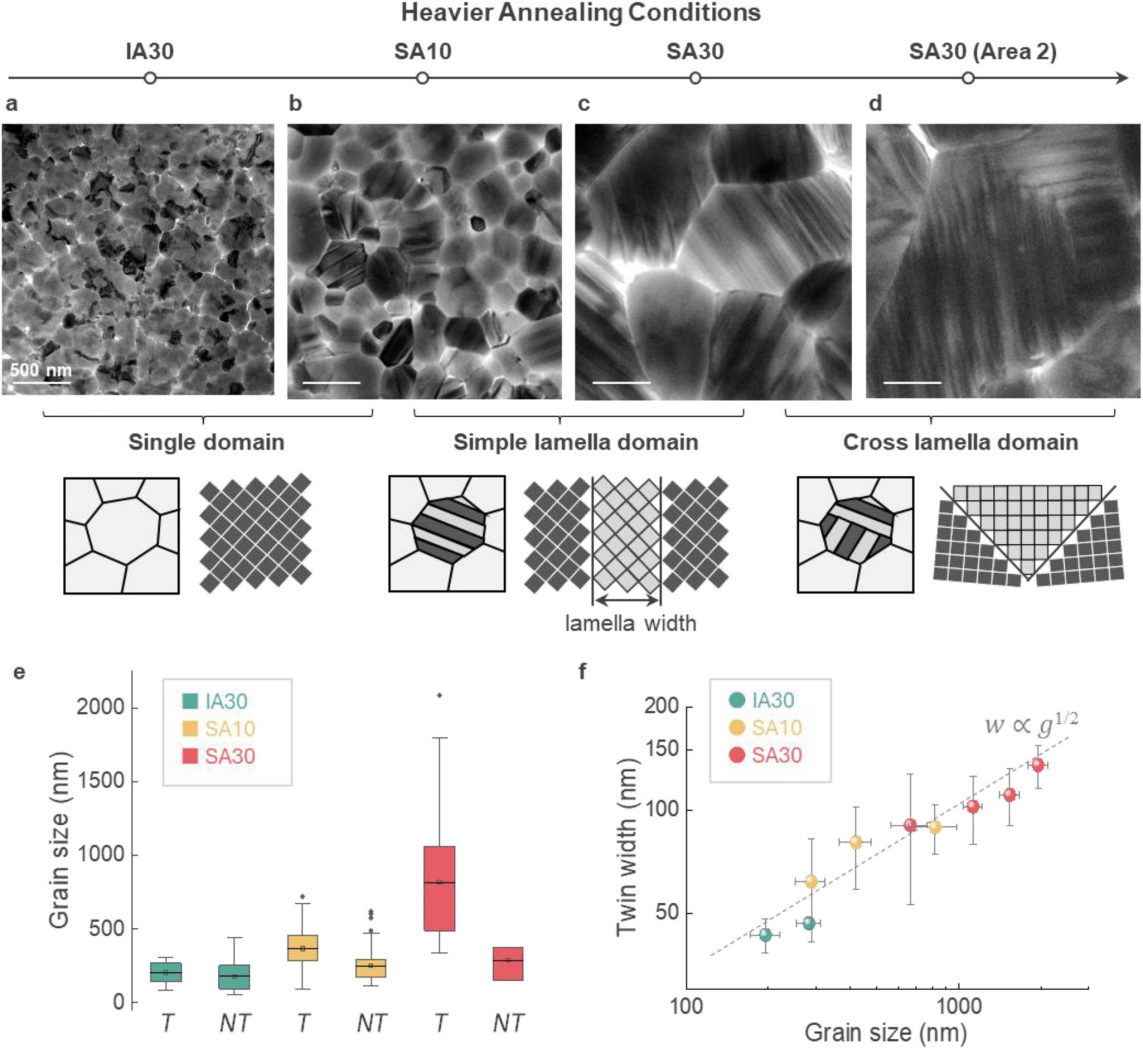
Effects of annealing on {112}_t_ ferroelastic twinning in MAPbI_3_ films. (a–d) BF‐TEM images of MAPbI_3_ films under different annealing conditions: (a) inert annealing for 30 min (IA30), (b) solvent annealing for 10 min (SA10), and (c,d) solvent annealing for 30 min (SA30). All scale bars, 500 nm. Schematics below illustrate single‐domain, simple‐lamella, and cross‐lamella configurations, in which the domain boundaries of both lamella types are {112}_t_ twin boundaries. (e) Size distribution of twinned (T) and non‐twinned (NT) grains for each annealing condition. (f) Log‐scale plot of twin lamella width (*w*) versus grain size (*g*), showing a square‐root dependence (*w* ∝ *g* ^1/2^; gray dashed line).

A wide range of grain sizes, from several tens of nanometers to a few micrometers, was achieved by varying the annealing duration (10 or 30 min) and atmosphere (inert gas or solvent vapor). Solvent vapor annealing is known to promote solvent‐assisted grain growth during annealing by enhancing ionic/precursor mobility and facilitating grain‐boundary migration, thereby producing larger grains within a given annealing time [53]. Three representative conditions were selected based on grain sizes, measured by scanning electron microscopy (SEM) for films deposited on indium‐doped tin oxide (ITO) substrates (Figure S4): IA30 (345 ± 130 nm, annealed in inert gas for 30 min), SA10 (550 ± 150 nm, solvent vapor for 10 min), and SA30 (950 ± 300 nm, solvent vapor for 30 min). We note, however, that TEM specimens prepared on carbon‐film grids from less concentrated precursor solutions exhibited a wider grain size distribution, often containing grains smaller than those in the SEM images. For example, in IA30 films, TEM revealed predominantly sub‐200 nm grains, contrasting with the 345 nm average grain size obtained from SEM measurements. XRD confirmed that annealing atmosphere did not significantly alter the crystalline structure or induce secondary phases (Figure S5).

Notably, the domain structures varied markedly with grain size associated with annealing conditions. In IA30 films (Figure 1a), internal lamellae were rarely detected, and most grains adopted a single‐domain configuration. By contrast, in SA10 and SA30, which contained predominantly >500 nm grains (Figure 1b–d), lamellae were frequently observed. As the grain size increased from IA30 to SA10 and SA30, both the prevalence of twinning and the spacing between lamellar stripes increased, along with a higher incidence of cross‐lamellar patterns (Figure S6). This size‐dependent twinning behavior is unlikely to arise directly from variations in the annealing atmosphere. Instead, it reflects the intrinsic effect of grain size, given that ferroelastic twinning occurs only after grain growth is complete—during the 330 K phase transition upon cooling from the 373 K annealing temperature [34]. Consistent with this interpretation, statistical analyses of twinned (T) and non‐twinned (NT) grains under fixed annealing conditions (Figure 1e) showed that twinned grains were larger than non‐twinned counterparts. This trend was particularly pronounced in SA30 samples, which exhibited a broad grain size distribution. These results support the intrinsic role of grain size in governing twinning behavior, while the influence of the annealing atmosphere is addressed separately below.

To understand these trends, we employed a thermodynamic equilibrium model for ferroelastic domain formation. The cubic‐to‐tetragonal phase transition induces mechanical stress in polycrystalline films due to constrained lattice deformation by adjacent grain clamping. Ferroelastic twinning relaxes this stress, with the equilibrium domain structure determined by the balance between twin wall formation energy and elastic strain energy (Note S4; Figures S7 and S8). Assuming isotropic clamping, the domain structure parameters are described by the following expressions: [54].

(1) $$w_{eq}={\left(\frac{\sigma g}{2kc{\left(t-1\right)}^{2}}\right)}^{1/2}$$

(2) $$g_{c}=\frac{\sigma }{2kc{\left(t-1\right)}^{2}}$$

Here, *w_eq_* is the equilibrium lamella width, *σ* the twin wall formation energy, *g* the grain size, *k* the proportionality factor, *c* the elastic constant, *t* the tetragonality, and *g_c_* ​ the critical grain size below which twinning does not occur.

To validate the model, we statistically extracted the lamella width (*w*) and grain size (*g*) from BF‐TEM images (n > 100 grains; Notes S2 and S3; Figures S9–S13; Table S1). A logarithmic plot of *w–g* relation in Figure 1f (with corresponding logarithmic scatter plot in Figure S11) shows a square‐root scaling behavior ($w\propto g^{\frac{1}{2}}$), consistent with Equation (1). Crucially, this scaling persisted across annealing conditions with small deviation from the expected trend. This result indicates that annealing conditions have minimal influence on the {112}_t_‐type ferroelastic twin formation, while they are indirectly linked through grain size. The critical grain size was experimentally determined to be ∼220 nm for MAPbI_3_ (see Figure 3a,b), which explains the observed absence of twinning (or single domain state) in smaller grains.

This agreement suggests that the observed domain structures are close to thermodynamic equilibrium. During cooling, domain walls pass through a high‐mobility stage just below the phase transition temperature [54, 55]. Natural cooling in our experiments likely allows sufficient time for domain walls to reach equilibrium configurations, resulting in proportionally spaced lamellae. However, reorienting domain walls, especially where two perpendicular lamella clusters meet, requires overcoming a significantly higher energy barrier. This explains the frequent appearance of crossing lamellae only in larger grains, where overcoming the higher energy barrier required for reorientation becomes increasingly difficult, despite their geometric instability (Notes S4.3 and S5; Figures S14 and S15). Controlling the cooling rate, thereby intentionally deviating the system from the equilibrium, may offer a route to modulate the density and distribution of {112}_t_ defects via domain growth kinetics [56, 57]. Furthermore, since ferroelastic domains can respond to external mechanical stress via domain wall motion and rearrangement to minimize total elastic energy [54, 58], domain structure may also be engineered through strain design. A more intrinsic approach is to manipulate the internal stress by engineering lattice anisotropy, wherein the extent of ferroelastic distortion, quantified by tetragonality (*c*/*a* ratio), acts as the thermodynamic driving force for twin formation [54].

Reducing tetragonality, by suppressing the primary thermodynamic driving force for ferroelastic twinning, can thus increase both domain width and the critical grain size for twinning, as predicted by (Equations 1 and 2). To examine this, we employed A‐site tuning by partially substituting MA with guanidinium (Gua), which is known to reduce tetragonality by preserving the 3D perovskite structure up to a 25% substitution level [59]. XRD results confirmed gradual reduction in tetragonality in MA_1‐x_Gua_x_PbI_3_ films with Gua substitution: for IA30, 1.0133 (Gua 0%), 1.0118 (Gua 5%), 1.0081 (Gua 10%), 1.0068 (Gua 15%), 1.0044 (Gua 20%), and 1.0025 (Gua 25%); and for SA30, 1.0133 (Gua 0%), 1.0113 (Gua 5%), 1.0076 (Gua 10%), 1.0050 (Gua 15%), 1.0034 (Gua 20%), and 1.0013 (Gua 25%) (Figure 4e; Figure S16). The systematic peak shift with Gua content confirms successful Gua incorporation into the perovskite lattice. Notably, under the SA30 condition, where grain size does not decrease with Gua content (Figure S17), this peak shift is gradual and accompanied by negligible peak broadening, indicating that Gua is relatively homogeneously distributed at the macroscopic scale, consistent with prior reports of well‐incorporated Gua up to 25% substitution [59]. Strain mapping by four‐dimensional scanning transmission electron microscopy nanobeam diffraction (4D‐STEM NBD) of representative <001>‐oriented Gua25 grains further supported this homogeneity at finer scales, showing that intragrain strain varied continuously across sub‐grain length scales, with only ∼0.5–1% variation (standard deviation) and no strongly strained regions indicative of pronounced local Gua segregation (Figure S32). Strain modulations of this magnitude are commonly observed in halide perovskites owing to their intrinsically soft, anharmonic lattice, and thus reflect the inherent structural dynamics of the perovskite framework rather than compositional inhomogeneity from local Gua segregation [20].

The combined effects of grain size and tetragonality on ferroelastic twin formation are shown in Figure 2. BF‐TEM images are arranged along two axes, grain size (or annealing condition; vertical) and tetragonality (or Gua concentration; horizontal), employing varied magnifications along the vertical axis. Tetragonality values plotted correspond to the average measured under IA30 and SA30 conditions at each Gua concentration (Figure 4e). For each Gua concentration, tetragonality values for IA30 and SA30 annealing conditions showed deviations within 0.001, indicating no significant effect of annealing on tetragonality in our experiments (Figure S16). Grain size of IA30 slightly decreased as Gua content increases but that of SA30 remained largely unchanged (Figure S17). Yellow dashed lines marked grains exhibiting discernible twinning. At high tetragonality (1.0133), twinning is observed across a wide range of grain sizes with the average domain width scaling with grain size, as described previously. As tetragonality decreased to 1.0116, twinning was suppressed in smaller grains (IA30) but persisted in larger grains (SA10, SA30). At 1.0059, twinning was mostly absent, appearing only in grains exceeding ∼2 µm (SA30).

**FIGURE 2:**
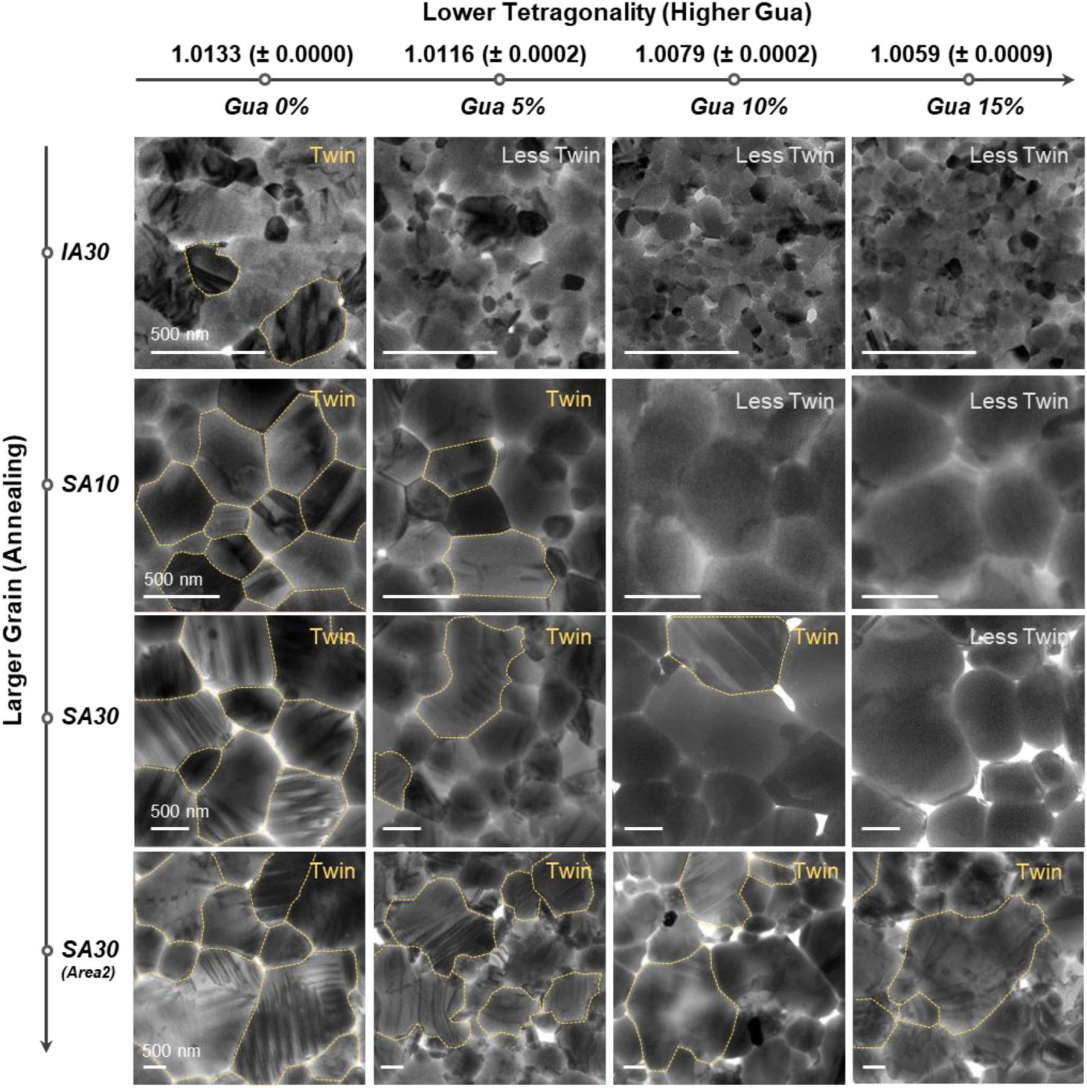
Effect of tetragonality and grain size on ferroelastic twinning in MA_1‐x_Gua_x_PbI_3_ perovskite films, observed via BF‐TEM. Tetragonality varies with Gua content (horizontal axis), and grain size with annealing conditions (vertical axis); average tetragonalities from XRD (IA30 and SA30 films) are shown for each Gua content. All scale bars, 500 nm (note the different magnifications along the vertical axis). Grains with discernible lamellar twins are outlined in yellow dashed lines; “Twin/Less Twin” labels at the top right indicate the extent of twinning in each image.

Twinning probability is quantified as a function of grain size at each tetragonality in Figure 3a (Note S6; Figure S18). The experimentally defined critical grain size at which twinning probability reaches 50% increased with decreasing tetragonality, reaching ∼1.7 µm at *t* = 1.0059. These values align with theoretical predictions derived from fitting Equation (2) to the experimental 220 nm value at 0% Gua (Figure 3b), though they are consistently higher, likely due to orientation‐dependent invisibility of twin boundaries (Note S3). This deviation can also arise from the reduced film continuity at high Gua content under SA30, where the grains tend to be more particle‐like, with pinholes between them, rather than densely packed, so that clamping is generally weaker and more heterogeneous. This looser clamping lowers the transformation stress transmitted between grains during the cubic‐to‐tetragonal transition, reducing the thermodynamic driving force for ferroelastic twinning and thereby further raising the apparent critical grain size relative to the isotropic‐clamping model (Equations 1 and 2). Consistent with this, the largest deviations are observed at Gua 10 and Gua 15, whose analyzed regions show lower grain‐packing density and film continuity than the other compositions (Figure S18b). Similarly, lamella widths measured in grains of 1 ± 0.1 µm closely match the theoretical model (Figure 3c).

**FIGURE 3:**
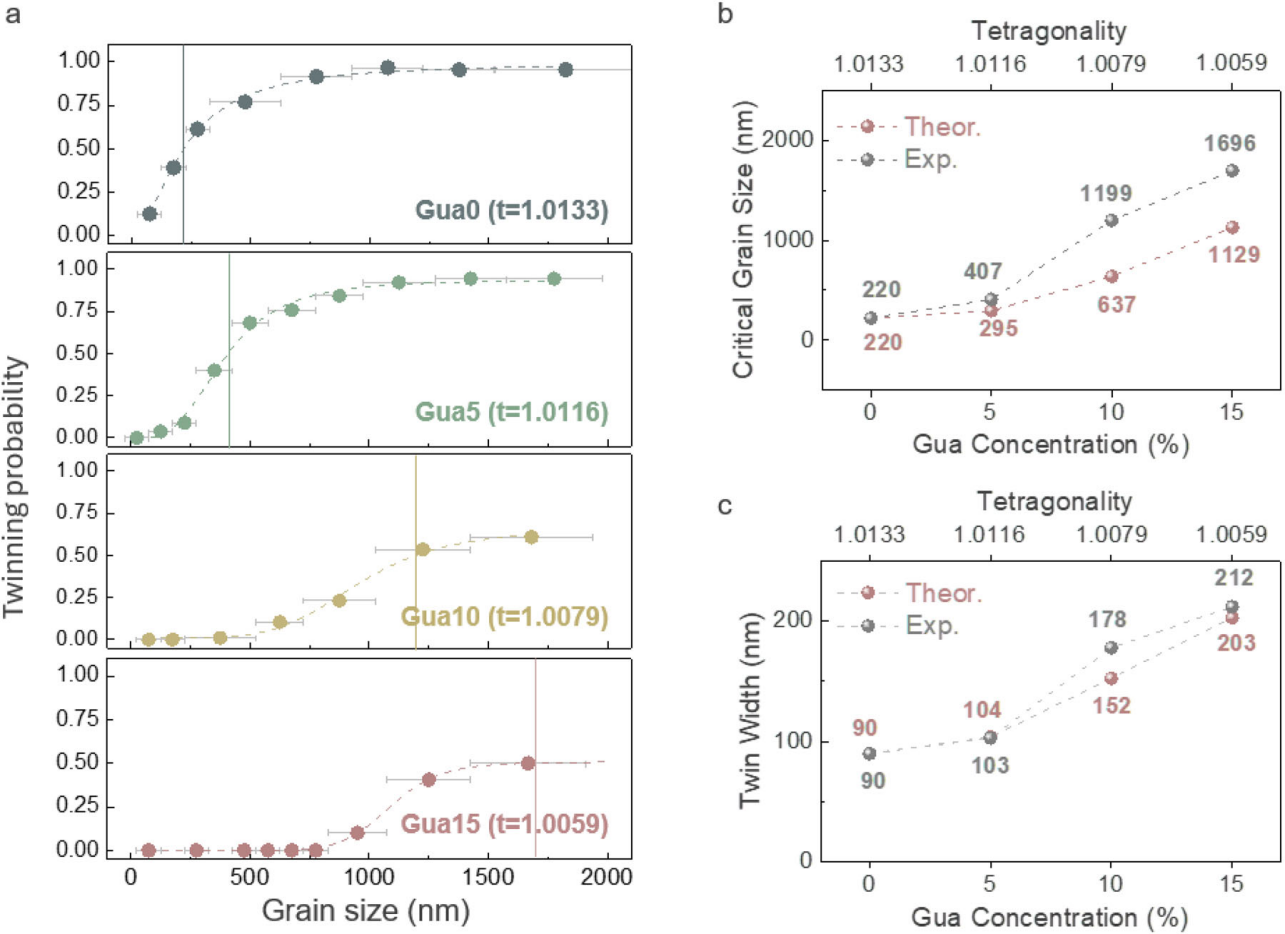
Statistics of ferroelastic twinning behaviors. (a) Twinning probability vs. grain size for films with 0%, 5%, 10%, and 15% Gua content, with average tetragonalities of 1.0133, 1.0116 (± 0.0002), 1.0079 (± 0.0002), and 1.0059 (± 0.0009), respectively (XRD, IA30 and SA30 films). Each point is the fraction of twinned grains within a grain‐size range (horizontal error bars); vertical lines mark the experimental critical grain size, where twinning probability reaches 50%. (b) Experimental and theoretical critical grain sizes (*g_c_*) vs. Gua content and tetragonality. Experimental *g_c_* are from panel a; theoretical values from fitting Equation (2), with parameters set by Gua 0% sample (*g_c_* = 220 nm). (c) Experimental and theoretical lamellar widths for grains of 1 ± 0.1 µm. Experimental widths are measured directly from TEM; theoretical widths from fitting Equation (1), with constants set by the Gua 0% film (lamellar width = 90 nm).

While the scaling laws offer a general framework for predicting ferroelastic twinning behavior, the quantitative applicability of (Equations 1 and 2) may vary across compositions of perovskite systems with different A‐ or X‐site ions, as the model parameters such as elastic constant and twin wall formation energy, are material‐specific. Previously reported FA_x_MA_1‐x_PbI_3_ films show no twinning below a tetragonality (*t*) of 1.004–1.013 (corresponding composition of 0.1 ≤ x ≤ 0.2; with *t* measured from electron diffraction) in around 300 nm grains [21]. In contrast, our MA_1‐x_Gua_x_PbI_3_ systems show no twinning below *t* ≈ 1.012 (with *t* measured from XRD) for about 300 nm grains. Despite possible variations in critical grain size or tetragonality threshold for twinning across compositions, the trend of increasing critical grain size with decreasing tetragonality is expected to hold broadly for mixed compositional systems of APbX_3_ (A = MA, FA, and Cs; X = I, Br, and Cl). This comparison suggests that the trend is primarily associated with tetragonality rather than being unique to Gua chemistry.

### 2.2 Formation Parameters of {111}_c_‐type Planar Defects

While moderate guanidinium (Gua) incorporation effectively suppresses {112}_t_‐type ferroelastic twins by reducing tetragonality, excessive Gua content (≥20%) induces another class of intragrain defects: {111}_c_‐type twins and stacking faults. Low‐dose BF‐TEM (Figure 4a) and selected‐area electron diffraction (Figure S19) identify the observed sharp boundaries as {111}_c_ planar defects, and atomic‐resolution HAADF‐STEM imaging (Note S7; Figures S20 and S21) directly visualizes nanodomains bounded by {111}_c_ defect planes (Figure 4b, left). The domain map reconstructed by combining inverse fast Fourier transforms (iFFTs) of distinct Bragg‐peak sets (Figure 4b, right), together with the Bragg‐filtered image (Figure 4c), resolves the twin boundaries and stacking faults, revealing a Pb‐sharing configuration consistent with prior predictions [31].

**FIGURE 4:**
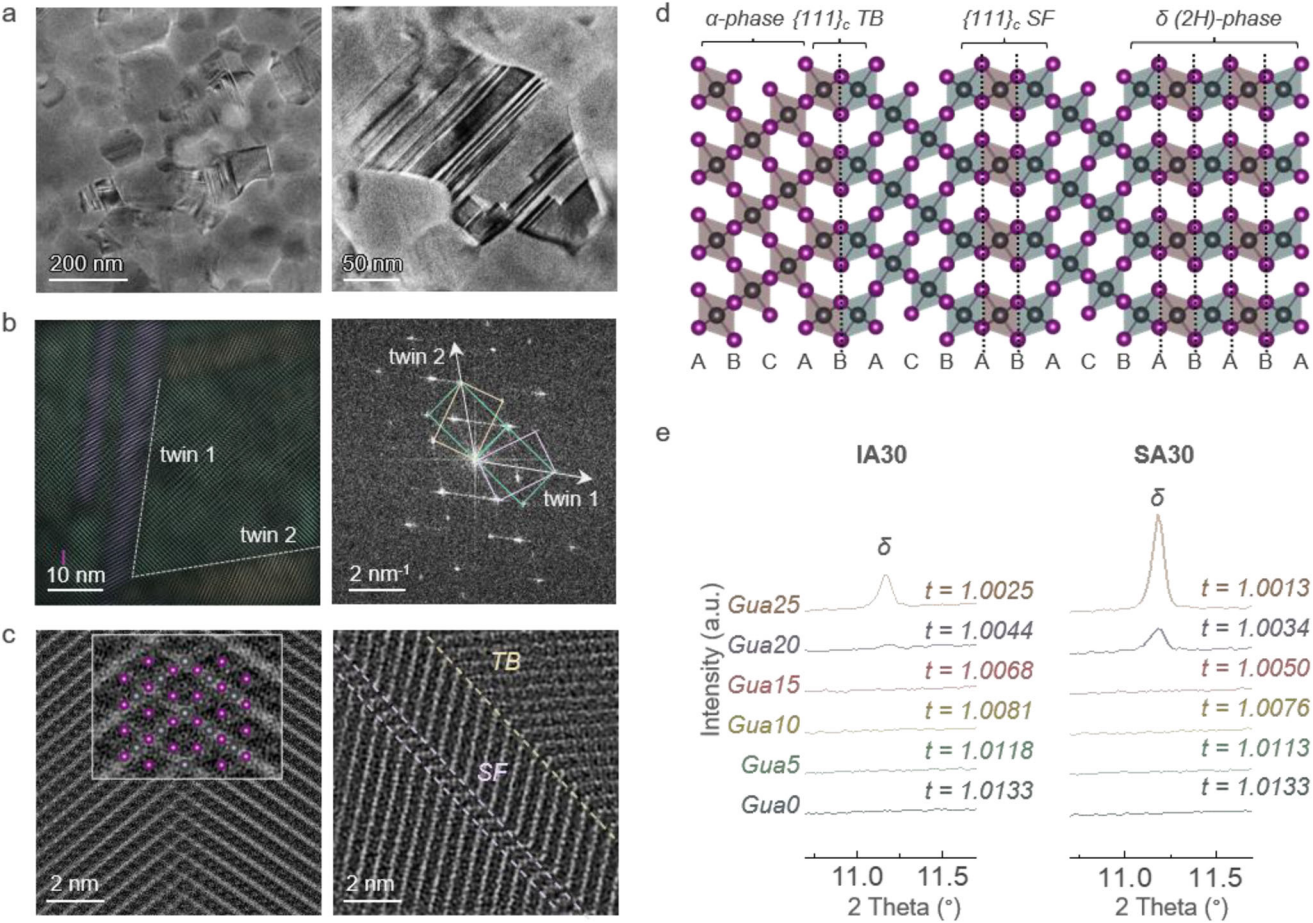
Tetragonality dependency of {111}_c_ planar defects. (a) BF‐TEM images of a high‐Gua film showing nanodomains bordered by {111}_c_ planar defects, at low (scale bar, 200 nm) and high (50 nm) magnification. (b) Colored map (left; 10 nm) from inverse FFT of the atomic‐resolution HAADF‐STEM image, resolving the {111}_c_ nanodomains into two twin variants (twin 1, twin 2); each domain corresponds to one set of diffraction spots outlined in the FFT (right; 2 nm^−1^). (c) Bragg‐filtered HAADF‐STEM images (scale bars, 2 nm). Left: lattice image with overlaid atomic model (inset). Right: a {111}_c_ twin boundary (TB) and a stacking fault (SF), marked by dashed lines. (d) Schematic atomic models of the α‐phase, TB, SF, and δ(2H)‐phase, with close‐packed stacking sequences below. (e) XRD patterns of MA_1‐x_Gua_x_PbI_3_ films (x = 0–0.25) near the δ‐phase reflection (∼11.2°) for IA30 (left) and SA30 (right) annealing, with tetragonality (*t*) values. The (S)TEM images in a–c were acquired from MA_0.8_Gua_0.2_PbI_3_.

XRD (Figure 4e) shows that, under both IA30 and SA30 annealing, films with Gua content ≥20% exhibit a new diffraction peak near 11.2°, corresponding to the hexagonal δ phase— a long‐range‐ordered polytype built from periodic {111}_c_ twinning of face‐sharing octahedra (e.g., 2H, 4H, depending on twinning sequence) [21, 60]. Because this reflection requires periodic face‐sharing stacking, isolated {111}_c_ defects do not produce it. However, both {111}_c_ planar defects and the δ phase originate from the same face‐sharing octahedral motif, so the structural driving force favoring one is likely to favor the other, as in SiC polytypes [61]. The growth of the δ‐phase peak with Gua content (Figure 4e) can therefore serve as indirect evidence that the {111}_c_ defect density also rises.

To directly quantify the {111}_c_ defect frequency, we used low‐dose 4D‐STEM NBD, since these defects are too densely and irregularly distributed, with boundaries too indistinct in BF‐TEM, for reliable per‐grain counting as done for {112}_t_ twins. From the scanning nanobeam diffraction dataset we reconstructed a virtual bright‐field image and segmented the grains (Note S8; Figure S24). Only grains exhibiting a {111}_c_ diffraction peak (*d* ≈ 3.66 Å) as a first‐order reflection together with a streak collinear with that peak were counted as defect‐bearing (representative patterns and ROI maps in Figures S25–S30). This peak‐plus‐collinear‐streak criterion works because streaking from face‐sharing stacking develops only along the hexagonal c‐axis (equivalent to the <111>_c_ direction of the 3C perovskite), whereas the *δ*‐phase reflections that fall near 3.66 Å either lie off this axis or appear only as higher‐order *c*‐axis reflections; neither can produce a first‐order 3.66 Å peak with a collinear streak. The criterion therefore selectively captures grains bearing {111}_c_ planar defects while excluding *δ*‐phase polytypes (Note S8); *δ*‐phase grains were instead identified separately (Figure S31, in part). On this basis (Figure 5), the defect‐bearing fraction was markedly higher for Gua25 than Gua20 in both number fraction (Gua20: 2.3 ± 1.9%; Gua25: 10.1 ± 3.5%) and the area fraction (Gua20: 3.3 ± 3.3%; Gua25: 13.9 ± 10.8%; mean ± standard deviation over four ROIs; Figure 5b) — consistent with the δ‐phase XRD reflection (Figure 4e), which is likewise more pronounced at 25% Gua.

**FIGURE 5:**
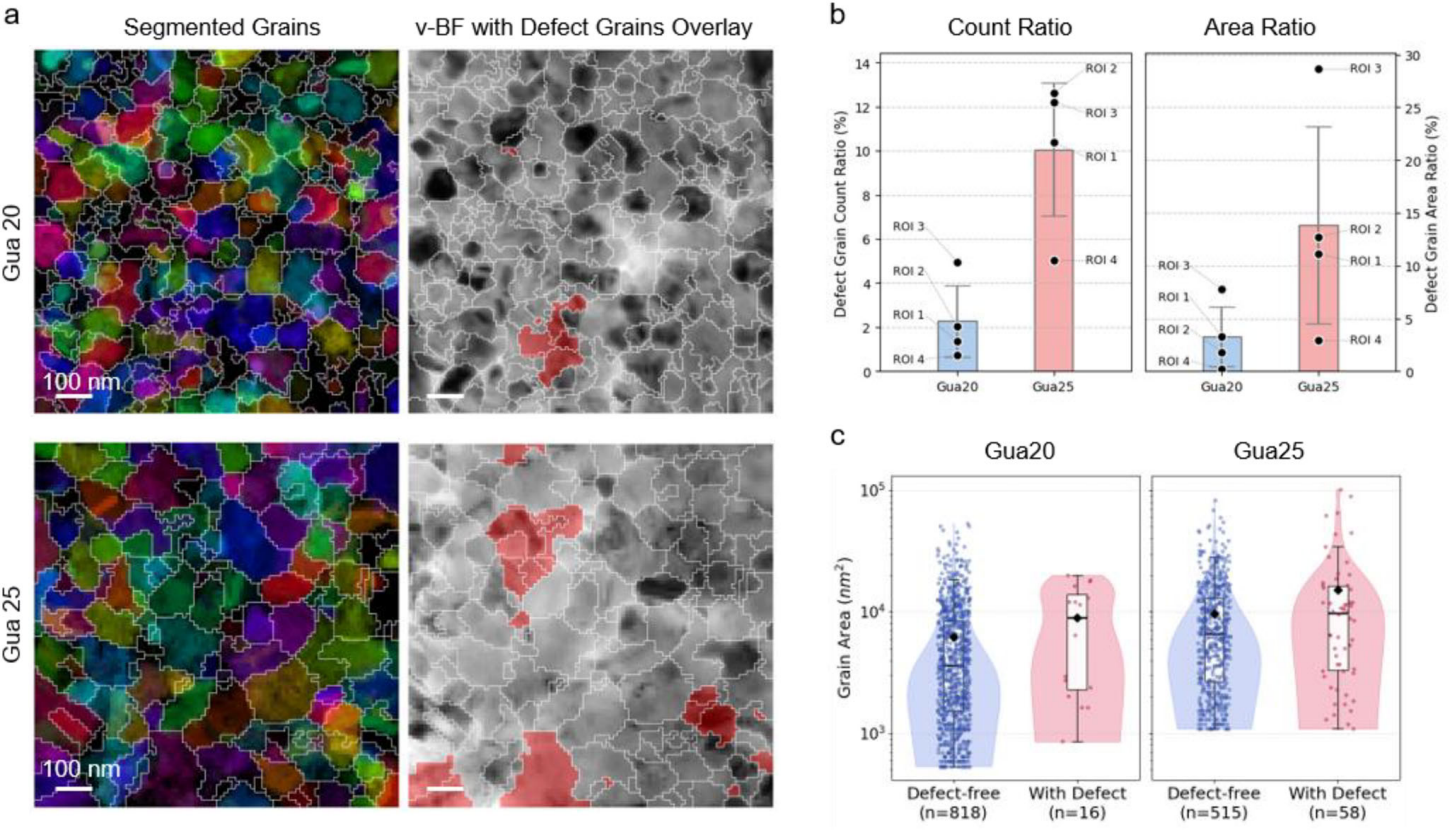
4D‐STEM quantification of defect‐bearing grains in Gua20 and Gua25 films. (a) Representative grain segmentation and defect‐grain identification for Gua20 (top) and Gua25 (bottom), shown for ROI1 of each composition; remaining ROIs (ROI 2–4), virtual BF overlays, and decomposed‐grain maps are in Figure S25 (Gua20) and S28 (Gua25). Left: real‐space grain map from NMF‐based segmentation (Figure S24, Methods), each grain in a distinct color. Right: virtual bright‐field image with defect‐bearing grains overlaid in red. Scale bars, 100 nm. (b) Fraction of defect‐bearing grains per film, as count ratio (defect‐grain count / total) and areal fraction (defect‐grain area / total area). Bars, mean over four ROIs; error bars, s.d.; points, individual ROIs (1–4). (c) Grain‐area distributions for defect‐free and defect‐bearing grains, pooled over all ROIs (n below each category). Diamonds, mean; points, individual grains; logarithmic area axis.

The emergence of these defects correlates with reduced tetragonality. Lower tetragonality can facilitate the formation of {111}_c_ boundaries, as the cubic phase can accommodate atomically coherent {111}_c_ twin planes, in contrast to the semi‐coherent (∼1° tilt) boundaries in tetragonal phases [31]. Density functional theory (DFT) calculations support this, showing {111}_c_ twin‐boundary formation energy decreasing with tetragonality (Figure S22). Consistently, the defect‐bearing grain ratio rises from Gua20 to Gua25 (IA30: tetragonality 1.0044 → 1.0025; Figure 5).

Defect‐bearing grains had a somewhat larger mean area than defect‐free grains (Figure 5c). However, because a grain is labeled defect‐bearing if even a single streak is observed, larger grains are inherently more likely to contain one. After correcting for this area‐dependent detection bias (size‐sampling bias), no statistically significant correlation remained between the occurrence of {111}_c_ defects and grain size (Note S9 and Figure S23). In other words, the larger mean size of defect‐bearing grains arises merely because larger grains are more likely to contain a defect, and is not evidence that {111}_c_ defects form preferentially in larger grains. This absence of a size effect reflects that {111}_c_ defects, unlike {112}_t_ twins, arise from stacking‐sequence errors during grain growth rather than from a post‐growth transformation; the critical‐grain‐size dependence seen for {112}_t_ twins is thus intrinsically absent here.

Annealing, by contrast, appears to influence the {111}_c_ defect density. Because our direct 4D‐STEM NBD quantification used IA30 films, the annealing dependence rests on XRD as indirect evidence: the δ‐phase peak is stronger for SA30 than IA30 (Figure 4e), and the integrated δ‐phase peak area (11.0°–11.5°), normalized to the perovskite {110}_t_ peak (13.9°–14.4°), was consistently larger in SA30. Since IA30 is the slower condition, this agrees with reports that slower crystallization suppresses δ‐phase formation [62]. The possible role of solvent vapor remains unclear, and the crystallization rate itself warrants further systematic study, but crystallization dynamics are broadly recognized as decisive for δ‐phase formation. For instance, vapor treatments with methylammonium or formamidinium thiocyanate convert δ‐phase into the photoactive α phase by promoting SCN^−^ adsorption and restructuring the octahedral network during crystallization [40]. Similarly, bulky ammonium acid additives such as 5‐ammonium valeric acid iodide and 5‐ammonium valeric acid chloride suppress δ‐phase emergence by slowing crystallization due to strengthened precursor interactions [62]. Collectively, these suggest that modulating crystallization — via annealing profiles or chemical additives — offers a promising route to suppress {111}_c_ planar defects. Ideally, this would reduce tetragonality while pushing the critical grain size toward the macroscale required for {112}_t_ twin formation, without inducing {111}_c_ defects.

### 2.3 Processing Window for Defect Suppression and Its Influence on Solar Cell Parameters

Based on our findings, we propose a processing window for minimizing intragrain planar defects, governed primarily by two factors: A‐site composition and annealing conditions. The formation of {112}_t_ twins is largely dictated by tetragonality and grain size, which are influenced by A‐site substitution and annealing parameters, respectively. Similarly, {111}_c_ defects show a strong correlation with reduced tetragonality, controlled by A‐site composition, and an apparent (XRD‐inferred) sensitivity to crystallization kinetics that could be tuned through annealing profiles (e.g., slower cooling). For the typical grain sizes of perovskite solar cells (<500 nm), both defect types can be suppressed when tetragonality is maintained within a processing window bounded by an upper threshold (*t*_upper_ ≈ 1.010) for {112}_t_ twin suppression and a lower threshold (*t_lower_* ≈ 1.005) for {111}_c_ defect suppression. The lower threshold for {111}_c_ defects is based on our experimental grain growth conditions (IA30 and SA30), and their density may be further reduced through careful control of crystallization kinetics, independent of tetragonality criteria. Beyond annealing, diverse approaches to modulate crystallization dynamics remain available, offering significant potential for further optimization. This window is grain‑size dependent: for larger defect‑free grains (∼1 µm), the upper tetragonality threshold tightens to *t*_upper_ ≈ 1.008, narrowing the permissible range.

To evaluate the effectiveness of this defect suppression approach, we fabricated solar cell devices with varying guanidinium (Gua) concentrations (*x* = 0, 0.05, 0.10, 0.15, 0.20, and 0.25) under a mild annealing condition (IA30). Figure 6a presents, on a common defect grain ratio axis, the fraction of {112}_t_‐twinned grains (from BF‐TEM images) and the fraction of {111}_c_ defect‐bearing grains quantified by 4D‐STEM NBD (IA30; Gua20 and Gua25). Time‐resolved photoluminescence (TRPL) and power conversion efficiency (PCE) measurements revealed maximum performance at 10%–15% Gua, coinciding with the lowest defect‐bearing grain ratios. Under these conditions we observed enhanced carrier lifetimes (Figure 6b and Figure S33; Table 1) and PCE values (Figure 6c; Table 2), along with improved photovoltaic parameters (Figures S34 and S35). Figure 6b shows the dependence of TRPL decay time constants (i.e., carrier lifetime) on the Gua content, where the fast decay time constant (τ_1_) and slow decay time constant (τ_2_) are attributed to surface/interface recombination and bulk recombination, respectively [57]. The slow time constant, τ_2_, exhibits a stronger correlation with the Gua content, and thus, with the twin density, as twins are located within the grain interiors. The sample containing 10%–15% Gua exhibits the longest carrier lifetime, which is consistent with reduced intragrain planar‐defect‐mediated recombination/transport losses. Consistent with this trend, the Urbach energy (E_U) extracted from the absorption edge decreases up to Gua15 (17.31 → 16.60 meV) but rises again at Gua20–25 (21.65–24.34 meV) (Table S2). Control experiments confirmed that these improvements were not driven by changes in bandgap (Figure S36), and no secondary phase was detected in the optimized 10%–15% Gua range (Figure S5). Although grain size decreased with increasing Gua content (Figure S17), the optimal performance at intermediate Gua levels (10%–15%), despite smaller grain sizes, suggests that planar‐defect density is the variable most closely tracking carrier transport within this fixed device series.

**FIGURE 6:**
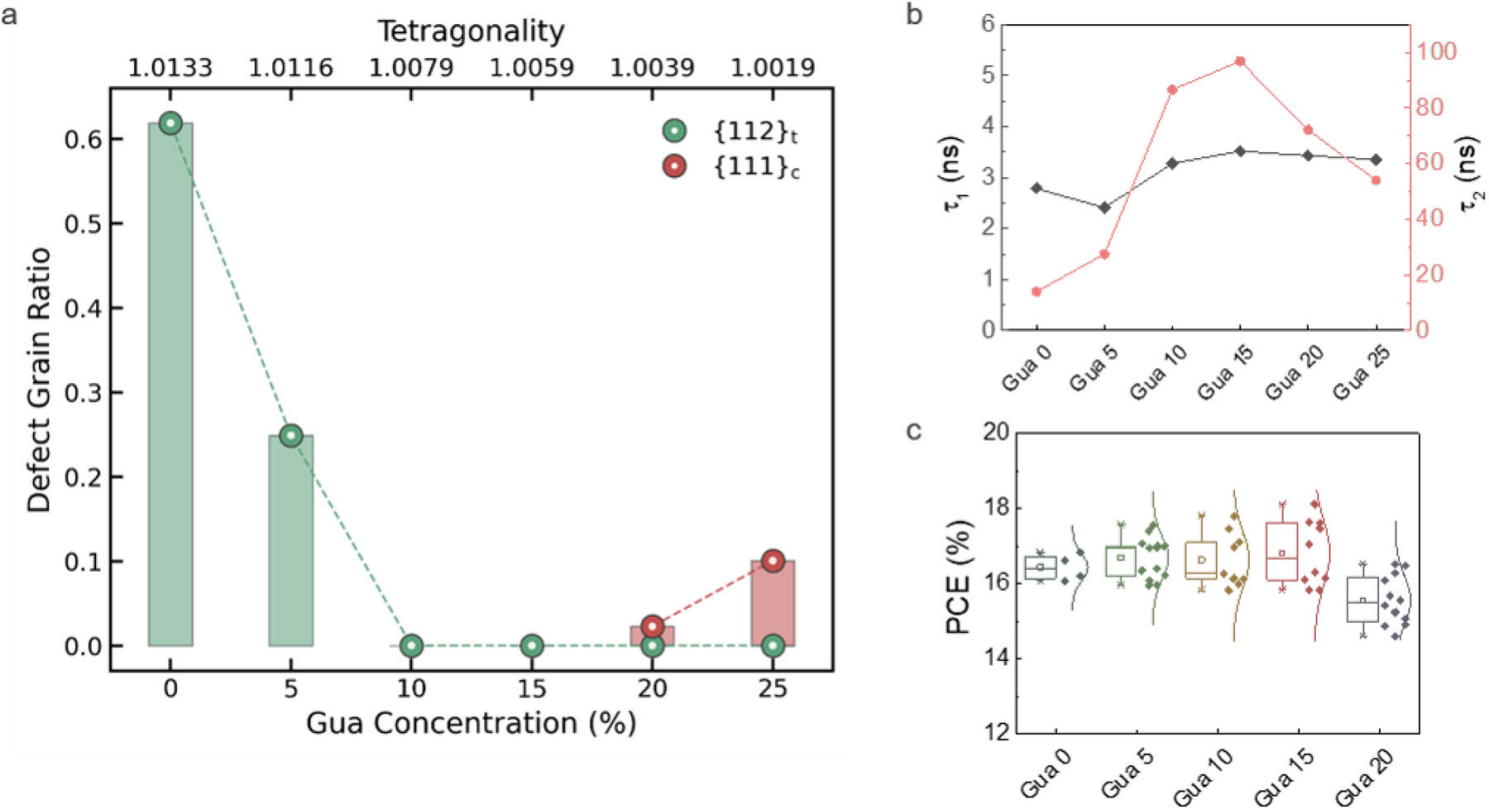
Correlation between planar defect occurrence and solar cell performance. All data are for MA_1‐x_Gua_x_PbI_3_ films under the IA30 annealing condition. (a) Defect‐bearing grain ratio (fraction of grains showing a given type of planar defects) versus Gua concentration, with XRD‐derived tetragonality on the top axis. Two populations share the left y‐axis: {112}_t_ ferroelastic twins from BF‐TEM (green) and {111}_c_ defects quantified by low‐dose 4D‐STEM NBD (orange; Gua20 and Gua25; see Figure 5 for full ROI statistics). (b,c) Fast (*τ_1_*) and slow (*τ_2_*) decay times from TR‐PL spectra (b) and power conversion efficiencies (c).

**TABLE 1:** Time‐resolved PL decay parameters.

	A_1_	*τ*_1_ (ns)	A_2_	*τ*_2_ (ns)	*τ*_avg_ (ns)
Gua 0	2554.90	2.79	12643.91	13.97	13.54
Gua 5	5381.79	2.88	10196.05	27.61	26.32
Gua 10	4431.99	3.28	8669.70	86.63	85.05
Gua 15	5871.71	3.52	6351.32	96.90	93.87
Gua 20	6347.83	3.43	5644.59	72.23	68.74
Gua 25	5624.17	3.36	6189.02	54.01	51.30

**TABLE 2:** Solar cells device parameters (best‐cell).

	*J*_sc_ (mA cm^−2^)	*V*_oc_ (V)	PCE (%)	FF
Gua 0	21.74	1.10	16.22	0.68
Gua 5	21.83	1.08	16.95	0.72
Gua 10	21.64	1.11	17.81	0.74
Gua 15	21.58	1.11	18.12	0.76
Gua 20	20.70	1.12	16.54	0.71

Beyond optimizing photovoltaic efficiency, ensuring long‐term device stability is essential for practical solar cell performance. While our compositional and annealing strategies effectively suppress intragrain planar defects, they do not inherently guarantee structural stability. For instance, cubic FAPbI_3_ readily transitions to the δ‐phase under humid conditions, leading to rapid performance degradation. In contrast, the tilted‐octahedra tetragonal phase has been proposed to offer greater resistance to such transitions [51, 63], but our findings show that it also promotes the formation of {112}_t_‐type defects. These trade‐offs highlight the need to design A‐site compositions and processing conditions that simultaneously minimize defect densities and stabilize the perovskite phase. In particular, because planar defects such as twin boundaries not only impede carrier transport but can also act as fast ion‐migration pathways that are detrimental to operational stability [21], suppressing these defects is expected to benefit both efficiency and operational durability. Future work should focus on identifying compositional and annealing regimes that achieve both low defect densities and high phase stability under operational conditions.

## 3 Conclusion

In this work, we systematically identified the formation parameters governing two dominant intragrain planar defects, {112}_t_ ferroelastic twins and {111}_c_‐type twins/stacking faults in model system MA_1‐x_Gua_x_PbI_3_ films. Using low‐dose TEM with independent control of A‐site composition and annealing conditions, we decoupled the roles of tetragonality, grain size, and crystallization kinetics. We show that {112}_t_ twin density scales with tetragonality and grain size but is insensitive to crystallization kinetics, whereas {111}_c_ defects are promoted by reduced tetragonality and yet remain independent of grain size.

From these findings, we establish a quantitative processing window in which both defect types are simultaneously suppressed, which is bounded by tetragonality thresholds of approximately 1.010 (upper, for {112}_t_ twins) and 1.005 (lower, for {111}_c_ defects) for submicron grains. Devices fabricated under these optimized conditions exhibit enhanced carrier lifetimes and power conversion efficiencies, supporting a correlation between planar‐defect suppression and improved photovoltaic performance. As phase stability can also depend on these structural parameters, future work should target compositions and processing protocols that jointly ensure low defect densities and robust stability under operational conditions.

Overall, this study provides practical design rules for minimizing intragrain planar defect formation by linking lattice anisotropy (tetragonality), grain size, and crystallization dynamics. These guidelines enable the rational tailoring of A‐site composition and crystallization conditions, offering a pathway to simultaneously enhance performance and stability in halide perovskite photovoltaics.

## 4 Methods

### 4.1 Perovskite Film Preparation

Perovskite precursor solutions were prepared by dissolving the desired stoichiometric composition ratio (MA_1‐x_Gua_x_PbI_3_, 0 ≤ x ≤ 0.25) of methylammonium iodide (MAI), guanidinium iodide (GuaI), lead iodide (PbI_2_) to give a concentration of 1.5 M in N,N‐dimethylformamide and dimethyl sulfoxide mixed solvent (9:1 v/v). Perovskite films were fabricated by spin coating the 1.5 M (for device fabrication) or 0.5 m (for TEM specimen preparation) precursor solution at 4000 rpm for 25 s, diethyl ether was dropped at 9 s as the antisolvent. Subsequently, substrates were annealed at 65°C for 1 min and 100°C for 30 min in N2 glove box.

### 4.2 Device Fabrication

The ITO substrates were cleaned using sonication with 2% Hellmanex solution, deionized water, acetone, and isopropyl alcohol, followed by ultraviolet ozone treatment for 15 min. A diluted SnO_2_ nanoparticle solution was spin‐coated onto glass/ITO substrates at 4000 rpm for 30 s. The ITO/SnO_2_ substrates were annealed at 150°C for 30 min in ambient atmosphere (R.T., RH < 40%). After cooling down, perovskite solution in anhydrous N,N‐dimethylformamide and dimethyl sulfoxide mixed solvent (9:1 v/v) containing MAI, GuaI, PbI_2_ for stoichiometric (MA_1‐x_Gua_x_PbI_3_, 0 ≤ x ≤ 0.25) was spin‐coated on ITO/SnO_2_ substrates at 4000 rpm for 25 s. The diethyl ether was dropped at 9 s, and then annealed at 65°C for 1 min and 100°C for 30 min. A PTAA solution consisting of 15 mg PTAA, 15 µL bis(trifluoromethane) sulfonamide lithium salt (Li‐TFSI) stock solution (170 mg Li‐TFSI in 1 mL acetonitrile), and 7.5 µL 4‐tertbutylpyridine in 1 mL toluene was spin‐coated on top of the perovskite layer at 3000 rpm for 30 s. Finally, 120 nm of Au electrode was deposited using thermal evaporation under 6.0 × 10^−6^ Torr.

### 4.3 Perovskite Film Characterizations

The morphology, grain size, and second phase formation of the materials were analyzed by field‐emission scanning electron microscopy (FE‐SEM, Merlin Compact, ZEISS). The absorbance and band‐gap energy of the films were identified using a UV–vis spectrometer (Cary5000, Agilent). The crystal phase, peak shift, and full width at half maximum (FWHM) were measured by X‐ray diffractometer (XRD, D8 ADVANCE, Bruker) using Cu Kα radiation (λ = 1.5418 Å). The PL spectra were obtained from spectrofluorometer with continuous 520 nm laser (FluoTime 300, PicoQuant). The ITO/perovskite films were excited from the front‐side to obtain the SSPL spectra. The emission wavelength corresponding to the SSPL peak maximum was then selected for the subsequent TRPL measurements. The obtained TRPL decay curves were analyzed using a bi‐exponential decay function, I(t) = A_1_ exp(−t/τ_1_) + A_2_ exp(−t/τ_2_). Here A_1_ and A_2_ represent the decay amplitudes, while τ_1_ and τ_2_ correspond to the fast and slow lifetime components, respectively. The average lifetime (τ_avg_) was determined using τ_avg_ = (A_1_τ_1_^2^ + A_2_τ_2_^2^)/(A_1_τ_1_ + A_2_τ_2_). The Urbach energy (E_U_) was extracted from the exponential absorption edge of the UV–vis spectra (Table S2)

### 4.4 Device Characterizations

The *J‐*‐*V* characteristics were measured under AM 1.5G illumination (100 mW cm^−2^) using a solar simulator (WACOM, class AAA). The active area was defined by the aperture size of the metal mask (0.14 cm^2^). The external quantum efficiency (EQE) spectra were obtained using an 85‐Hz‐chopped monochromatic light without light or potential bias (QUANTX‐300, Newport). The *J–V* and EQE data were acquired on the same device; the EQE‐integrated *J*sc (20.16 mA cm^−^^2^) was ∼6.6% lower than the *J–V* value (21.58 mA cm^−^^2^), which we attribute to partial photocurrent loss during the prolonged, sequential‐wavelength EQE acquisition, given the light‐, oxygen‐, moisture‐, and heat‐sensitive MAPbI_3_/PTAA. The electrochemical impedance spectroscopy (EIS) and thermal admittance spectroscopy (TAS) were measured in dark conditions using a potentiostat (XENNIUM, ZAHNER) (Figure S37).

### 4.5 TEM Specimen Preparation

To prevent degradation of metal halide perovskite films during TEM specimen preparation, commonly caused by ion beam damage or mechanical stress, we adopted a direct spin‐coating method onto thin carbon film‐supported Cu TEM grids, as previously reported [34, 43]. This approach not only minimizes potential damage during sample preparation but also yields a large field of view, enabling statistical analysis of sub‐grain microstructures. While the carbon film substrate differs from those typically used in device fabrication (e.g., indium tin oxide, ITO), potentially resulting in variations in grain size or morphology, we carefully selected TEM regions that exhibited morphological features similar to those observed on ITO‐coated films in SEM analysis.

### 4.6 TEM Characterization

Bright‐field TEM imaging and selected‐area electron diffraction (SAED) were conducted using a 200 kV field‐emission TEM (Tecnai F20, Thermo Fisher Scientific) at the Research Institute of Advanced Materials (RIAM), Seoul National University. All TEM images and diffraction patterns were acquired from previously unexposed areas of the specimen, except where both were intentionally collected from the same location; unless otherwise noted, the accumulated electron dose for BF‐TEM and SAED was kept below 1 e^−^ Å^−^^2^. Atomic‐resolution high‐angle annular dark‐field STEM (HAADF‐STEM) was performed at room temperature using a spherical aberration‐corrected STEM (JEM‐ARM 200F, JEOL Ltd., Tokyo, Japan) equipped with a cold field‐emission gun (cold‐FEG) at the National Center for Inter‐University Research Facilities (NCIRF), Seoul National University, operated at 200 kV with a semi‐collection angle range of 80–340 mrad. Images were acquired with a spot size of 10C, a cold‐FEG emission current of 1 µA, a dwell time of 4 µs, and a frame resolution of 4096 × 4096 pixels.

### 4.7 4D‐STEM Nanobeam Diffraction (NBD)

Four‐dimensional STEM (4D‐STEM) was performed in NBD mode on a double aberration‐corrected STEM (Themis Z, Thermo Fisher Scientific) equipped with an electron microscope pixel array detector (EMPAD) at RIAM, Seoul National University. A near‐parallel nanobeam (convergence semi‐angle 0.1 mrad) was used with a beam current of 5 pA, a 256 × 256 pixel diffraction‐pattern frame, a real‐space scan step of 16.116 nm, and a 1 ms per‐pixel dwell time, corresponding to an accumulated dose of ∼1 e^−^ Å^−^^2^; a diffraction pattern was recorded at each scan position, and four regions of interest (ROIs) were acquired per composition (Gua20 and Gua25). After masking the direct beam and binarizing the diffraction patterns (so that only peak positions were used as features), non‐negative matrix factorization (NMF) was applied, followed by separation and merging of the resulting components to construct a cleaned grain label map (see Note S8 and Figure S24). Each grain was then classified as defect‐bearing or defect‐free from its representative NBD pattern (Figures S26 and S29): a grain was counted as {111}_c_‐defect‐bearing only when its pattern showed a {111}_c_ diffraction peak together with a streak along the same direction, whereas patterns assignable to δ‐phase polytypes were excluded from the count and summarized separately in Figure S31. The number (count) ratio and areal fraction of defect‐bearing grains were then computed per ROI and averaged. The grain‐segmentation and classification code (GitHub), together with the raw 4D‐STEM datasets and the complete set of per‐ROI results (Zenodo), is openly available, as detailed in the Data Availability Statement.

### 4.8 Intragrain Strain Mapping

Local strain within individual Gua25 grains was mapped from the same 4D‐STEM NBD datasets using the open‐source py4DSTEM package. These strain‐mapping NBD data were acquired at 100 kX magnification with a large camera length of 180 cm. A single grain was first isolated by the NMF‐based segmentation described above. To robustly localize the reciprocal‐lattice reflections, the autocorrelation of each nanobeam diffraction pattern was computed, and reflection positions were detected by template (cross‐correlation) matching with subpixel refinement; the diffraction origin was then measured and corrected for scan drift. Two basis vectors (*g*_1_, *g*_2_) were fitted across the grain, and the in‐plane strain tensor (*ε_xx_, ε_yy_, ε_xy_*) together with the lattice rotation (*θ*) was computed relative to a reference lattice taken from the grain interior, with scan positions lacking the reference reflections excluded. The full strain‐analysis code is openly available on GitHub, as detailed in the Data Availability Statement.

### 4.9 Density Functional Theory (DFT) Calculations

DFT calculations were performed using the Vienna Ab initio Simulation Package (VASP). A projector‐augmented wave (PAW) method was used for interactions between the valence electrons and the ionic cores. The generalized gradient approximation (GGA) in the Perdew–Burke–Ernzerhof (PBE) formulation was used for the exchange–correlation functional. The electron wave functions were expanded in a plane‐wave basis set with a kinetic energy cutoff at 520 eV. The {111}_c_ twin boundary models with 144 atoms (Figure S22b), with a Γ‐centered 3 × 3 × 1 k‐point set, were relaxed with fixed unit cell parameters until the residual forces were below 0.01 eV Å^−1^. The unit cell parameters were varied to control tetragonality while keeping the stabilized unit‐cell volume fixed (Figure S22c). The convergence threshold for the self‐consistent field (SCF) iteration was set at 10^−7^ eV.

## Author Contributions

**Jin Young Kim**: conceptualization, funding acquisition, Writing – review and editing, project administration, resources, supervision, validation, investigation, methodology, formal analysis. **Byeongjun Gil**: conceptualization, investigation, writing – original draft, methodology, validation, visualization, writing – review and editing, software, formal analysis, data curation. **So Jeong Park**: data curation, software, formal analysis, methodology, visualization, validation, writing – review and editing, conceptualization, investigation. **Miyoung Kim**: supervision, resources, project administration, writing – review and editing, writing – original draft, conceptualization, funding acquisition, methodology, validation, investigation, formal analysis.

## Artificial Intelligence (AI) Statement

During the preparation of this manuscript, the authors used AI‐based tools for two purposes. Claude (Anthropic) and Antigravity (Google) were used to assist in developing the code for grain segmentation, defect classification, and strain mapping used in the data analysis (openly available as described in the Data Availability Statement). ChatGPT (OpenAI) and Claude (Anthropic) were used to improve the language and readability of the text. All AI‐assisted content, including the generated code, was reviewed, tested, and verified by the authors, who take full responsibility for the content of this publication. AI tools were not used to generate, interpret, or fabricate the underlying research data or results.

## Funding

This work was supported by the National Research Foundation of Korea (NRF) grants funded by the Korea government (MSIT) (RS‐2022‐NR070586, RS‐2026‐25540195, and RS‐2023‐00273532).

## Ethics Statement

This study did not involve human participants, human‐derived samples, or animal subjects. Therefore, ethics approval was not required.

## Conflicts of Interest

The authors declare no conflicts of interest.

## Supporting information




**Supporting File**: advs77365‐sup‐0001‐SuppMat.docx.

## Data Availability

The data that support the findings of this study are available in the article and its Supporting Information. The grain‐segmentation and {111}_c_ defect‐classification code, and strain mapping code are openly available at https://github.com/ByeongjunGil/GrainSTEM, and the raw 4D‐STEM nanobeam diffraction datasets for all regions of interest are archived at Zenodo (https://doi.org/10.5281/zenodo.21025176). Any further data are available from the corresponding author upon reasonable request.
