# Supplementary material for: Design Rules for Quantitative Control of Intragrain Planar Defects in Halide Perovskites
Source: Adv Sci (Weinh). 2026 Aug 24:e77365. Online ahead of print. doi: 10.1002/advs.77365 (PMC13502282; doi:10.1002/advs.77365)
Supplement: Supplementary file 1 [file ADVS-9999-e77365-s001.docx]

**Supporting Information for:**

**Design Rules for Quantitative Control of Intragrain Planar Defects in Halide Perovskites**

*Byeongjun Gil*^†^*, So Jeong Park*^†^*, Jin Young Kim***, Miyoung Kim**

B. Gil

Department of Materials Science and Engineering, Seoul National University, Seoul 08826, Republic of Korea.

S. J. Park

Department of Materials Science and Engineering, Seoul National University, Seoul 08826, Republic of Korea.

Research Institute of Advanced Materials (RIAM), Seoul National University, Seoul 08826, Republic of Korea.

J. Y. Kim

Department of Materials Science and Engineering, Seoul National University, Seoul 08826, Republic of Korea.

Research Institute of Advanced Materials (RIAM), Seoul National University, Seoul 08826, Republic of Korea.

School of Transdisciplinary Innovations, Seoul National University, Seoul 08826, Republic of Korea.

E-mail: jykim.mse@snu.ac.kr

M. Kim

Department of Materials Science and Engineering, Seoul National University, Seoul 08826, Republic of Korea.

Research Institute of Advanced Materials (RIAM), Seoul National University, Seoul 08826, Republic of Korea.

E-mail: mkim@snu.ac.kr

Funding:

This work was supported by the National Research Foundation of Korea (NRF) grants funded by the Korea government (MSIT) (RS-2022-NR070586, RS-2026-25540195, and RS-2023-00273532).

Keywords:

Halide perovskites, intragrain planar defects, low-dose transmission electron microscopy, defect engineering, perovskite solar cells

B. Gil and S. J. Park contributed equally to this work.

**Contents**

**Supporting Note List**

Note S1. Electron irradiation conditions for observing fragile ferroelastic domain structures

Note S2. Measurement of average lamella width as a function of grain size

Note S3. Orientation-dependent ferroelastic twin contrast and validation of statistical analysis

Note S4. Origin of ferroelastic twin domain formation in tetragonal perovskites

Note S5. Angular misfit boundaries in crossing-type lamella structures

Note S6. Measurement of ferroelastic twinning probability with respect to grain size

Note S7. Low-dose atomic-resolution scanning transmission electron microscopy

Note S8. 4D-STEM NBD data processing methodology

Note S9. Area-dependent detection bias in the 4D-STEM defect-grain statistics

**Supporting Figure List**

Figure S1. Electron beam-induced degradation of perovskite lattice structures

Figure S2. Electron beam damage experiments for intragrain planar defect structure observation

Figure S3. Selected-area diffraction patterns of {112}ₜ ferroelastic twins in MAPbI_3_

Figure S4. Grain size distributions under different annealing conditions

Figure S5. X-ray diffraction patterns of MA_1-x_Gua_x_PbI_3_ films under IA30 and SA30 conditions

Figure S6. Grain size dependence of the evolution of crossing-type lamellae

Figure S7. Ferroelastic twin stripe contrast in isolated grains

Figure S8. Schematic of ferroelastic twin domain formation mechanism

Figure S9. Schematic for measuring the relationship between lamella width and grain size

Figure S10. Lamella width versus grain size from BF-TEM of MAPbI_3_ films

Figure S11. Log–log plot of lamella width versus grain size

Figure S12. Visibility of ferroelastic domains in BF-TEM under low-dose conditions

Figure S13. Tilt-series BF-TEM of ferroelastic twin domain structures

Figure S14. Crystallographic configurations of {112}ₜ twin wall orientations

Figure S15. Schematic of angular misfit at crossing-type lamella boundaries

Figure S16. Tetragonality and unit cell parameters of MA_1-x_Gua_x_PbI_3_ from XRD

Figure S17. Grain sizes as a function of Gua concentration in MA_1-x_Gua_x_PbI_3_ films.

Figure S18. BF-TEM images of MA_1-x_Gua_x_PbI_3_ films under IA30 and SA30

Figure S19. Selected-area diffraction patterns of {111}_c_ planar defects in MA_0.8_Gua_0.2_PbI_3_

Figure S20. Electron beam tolerance of MA_1-x_Gua_x_PbI_3_ for atomic-resolution STEM

Figure S21. Atomic-resolution HAADF-STEM images of MA_0.8_Gua_0.2_PbI_3_ perovskites

Figure S22. DFT-calculated formation energy of {111}_c_ twins as a function of tetragonality

Figure S23. Grain size effects on the formation of {111}_c_ planar defects

Figure S24. 4D-STEM NBD workflow for grain segmentation and defect-grain classification

Figure S25. Grain-by-grain 4D-STEM NBD defect quantification for Gua20 (MA_0.8_Gua_0.2_PbI_3_)

Figure S26. Representative per-grain NBD patterns (ROI1) for Gua20

Figure S27. {111}_c_-oriented streaks in the grain-by-grain diffraction patterns of Gua20

Figure S28. Grain-by-grain 4D-STEM NBD defect quantification for Gua25 (MA_0.75_Gua_0.25_PbI_3_)

Figure S29. Representative per-grain NBD patterns (ROI1) for Gua25

Figure S30. {111}_c_-oriented streaks in the grain-by-grain diffraction patterns of Gua25

Figure S31. Identification and distribution of the δ phase

Figure S32. Intragrain strain distribution in Gua25 from 4D-STEM NBD

Figure S33. Time-resolved PL spectra of the perovskite absorber layers with varied Gua content.

Figure S34. Photovoltaic performance of single-junction devices with varied Gua content

Figure S35. EQE spectra of optimized perovskite solar cells with different Gua ratios

Figure S36. Optical bandgap estimation from Tauc plots of Gua-containing films

Figure S37. EIS spectra of MA_1-x_Gua_x_PbI_3_ solar cells

**Supporting Table List**

**Table S1.** Statistics of lamella domain wall intersections versus grain size in MAPbI_3_

**Table S2.** Urbach energy (E_U_) of MA_1-x_Gua_x_PbI_3_ perovskite films.

**Note S1. Electron irradiation conditions for observing fragile ferroelastic domain structures**

Metal halide perovskites are highly susceptible to electron beam damage, with their crystal structure rapidly degrading into secondary phases such as PbI_2_ and Pb under exposure.^[1]^ This damage can be especially severe in defect regions, which are inherently less stable than the bulk.^[2]^ To identify beam conditions that preserve defect structures, we conducted preliminary dose-dependent experiments.

Among parameters influencing beam damage—accelerating voltage, temperature, flux, and total dose—prior studies identify total dose as the dominant factor. Accordingly, we evaluated structural degradation using selected area diffraction pattern (SADP) analysis across a range of accumulated doses. In MAPbI_3_ films, the {110}ₜ peak intensity dropped by half at ~30–50 e⁻ Å⁻^2^ but remained stable below 10 e⁻ Å⁻^2^ (Figure S1), indicating structural preservation below this threshold.

However, ferroelastic domains were disrupted even at lower doses: lamellar features began deforming at ~3.5 e⁻ Å⁻^2^ and nearly disappeared by ~5.9 e⁻ Å⁻^2^ (Figure S2a), in contrast to {111}c planar defects that remained intact at much higher doses (Figure S2b). This heightened sensitivity arises from the strain-driven nature of ferroelastic domains, where beam-induced atomic displacement provides an alternative relaxation pathway, destabilizing the balance between stress and wall formation energy. Additionally, the small (~1°) lattice mismatch across twins implies a low energy barrier for domain merging. Such deformation hampers accurate measurement of lamellar width and determination of the critical grain size. To avoid this, we limited the total dose to below 1 e⁻ Å⁻^2^ for domain imaging.

**Note S2. Measurement of average lamella width as a function of grain size**

To statistically analyze the relationship between lamella width and grain size, lamella width was measured in terms of grain size from BF TEM images. Since twin domain features vary significantly depending on the orientation, as noted in Note 3, the measurement of lamella domain width can introduce errors. In particular, the sharpness of the domain wall changes according to the relationship between the twin plane and the optic axis, and this unclear boundary can greatly affect the accuracy of domain width measurements. Therefore, as shown in Figure S9, we measured the average lamella width by drawing a line perpendicular to the domain wall (normal length) and dividing this length by the number of intersections with the domain wall plus one. Grain size was calculated as the average of the normal length and the parallel length. This approach is used because even if the twin plane has an angular deviation from the optic axis causing the wall to become blurred, the number of contrast reversals remains equal to the number of walls.

$$Average Lamella Width = \frac{Normal Length}{Number of Intersection+1}$$

$$Grain Size= \frac{Normal Length+Parallel Length}{2}$$

To improve the accuracy of domain width measurements, we performed width measurements only on grains where intersections could be clearly identified, among the many grains showing twin contrast. More than one hundred grains were investigated as provided in Figure S10. The measured parallel length, normal length, and number of intersections for each index are provided in Table S1. The log-scale scatter plot data showing the relationship between width and grain size, calculated from these measurements, is presented in Figure S11.

**Note S3. Orientation-dependent ferroelastic twin contrast and validation of statistical analysis**

Twin domains in a crystallographic relationship can exhibit distinct intensities in TEM bright-field (BF) images, enabling the visualization of domain structures. However, their visibility strongly depends on the crystallographic orientation. Specifically, when the objective aperture is centered on the optic axis, twin domains are not observable if the twin plane is exactly perpendicular or parallel to the optic axis.

If the twin plane is perpendicular to the optic axis (i.e., aligned along the beam direction), intensities from each domain overlap in depth, making them indistinguishable. For this reason, {112}ₜ twins are not visible when the {112}ₜ normal vector aligns with the zone axis. Conversely, if the twin plane is parallel to the optic axis, although there is no overlap along the beam path, the electron transmission through each domain remains identical because the diffraction intensity ratios are the same. This is the case for the <1̅10>ₜ zone axis, where {112}ₜ twins also become unobservable.

Thus, domain contrast in BF TEM appears only when the twin plane is neither perfectly parallel nor perpendicular to the optic axis. Within this angular range, twin contrast varies with orientation. When the twin plane is nearly parallel to the optic axis, the domain wall appears sharp; when nearly perpendicular, the wall becomes blurred or indistinct. For example, {112}ₜ twins are invisible along the exact <1̅10>ₜ axis but become sharply defined with small angular tilts, then gradually lose contrast as the deviation increases. These variations are illustrated in Figure S12, which shows a range of domain wall appearances.

Due to this orientation dependence, caution is required when identifying and quantifying twin domains. In particular, the absence of contrast does not necessarily indicate the absence of twinning. As shown in Figure S13, TEM images of the same MAPbI_3_ grain at tilt angles of 0°, 2°, and 4° demonstrate how domain contrast changes with orientation. The boxed grain shows clear domains at 0° and 2° but none at 4°, highlighting that domain invisibility can result from geometric alignment rather than structural absence.

As a result, estimates of twinning probability based solely on the observed fraction of twinned grains may underestimate the true value. Nonetheless, because the observed and actual probabilities are proportional—assuming no significant differences in their preferred orientations of films (as supported by XRD in Figure S5)—relative comparisons between conditions remain valid.

**Note S4. Origin of ferroelastic twin domain formation in tetragonal perovskites**

Ferroelastic domain structures emerge upon a symmetry-lowering phase transition from a paraelastic to a ferroelastic phase. While many ferroelastic phases are concurrently ferroelectric, some exhibit purely ferroelastic behavior. In tetragonal metal halide perovskites such as MAPbI_3_, this transition occurs from a cubic (Pm3̅m) to a tetragonal phase. Two space groups have been proposed for the low-symmetry phase—I4/mcm (purely ferroelastic) and I4/cm (both ferroelastic and ferroelectric)—though their distinction remains under debate. Irrespective of this ambiguity, ferroelasticity is unambiguously observed, whereas ferroelectricity remains inconclusive. In our study, the system is treated as purely ferroelastic, and domain configurations are analyzed by considering mechanical stress energy alone, excluding electrostatic effects such as depolarization fields. This assumption is justified by prior studies on analogous systems, including BaTiO_3_, where mechanical contributions dominate ferroelastic domain formation.

**S4.1. Thermodynamic Considerations of Ferroelastic Domain Formation**

In MAPbI_3_, ferroelastic twinning occurs along the {112}ₜ mirror planes. These twin walls are energetically unfavorable relative to the bulk, and in the absence of external driving forces, a single-domain configuration would represent the equilibrium state. However, mechanical stress generated during the cubic-to-tetragonal phase transition provides a thermodynamic driving force for twin formation. Various sources of this stress have been proposed, including epitaxial strain from substrate,^[3]^ clamping from adjacent grains,^[4]^ and surface-related effects such as dead layers or surface tension.^[5]^

In polycrystalline films, the adjacent grain clamping model proposed by Arlt offers a compelling explanation^[4]^. The transition induces anisotropic lattice distortions—particularly along the c-axis—that alter crystallite shape. In a densely packed polycrystalline system, neighboring grains constrain these shape changes, leading to stress accumulation at grain boundaries. Formation of {112}_t_ twin domains relieves this stress by restoring partial symmetry through mirror operations, effectively compensating for the symmetry loss during the phase transition. As illustrated by the lattice model in Figure S8e, this symmetry recovery lowers the overall stress energy. However, since twin walls are intrinsically less stable than the bulk, increasing wall density also raises the total system energy. The equilibrium domain width is therefore governed by a balance between stress energy (Equation S1) and twin wall formation energy (Equation S2), with equilibrium achieved when the two are equal (Equation 1 and 2 in the main text). This framework captures the observed dependence of domain width on both grain size and tetragonality.

$$Stress energy = \frac{2kc{w(t-1)}^{2}}{g}$$

(S1)

$$Twin wall formation energy= \frac{\sigma}{g}$$

(S2)

Where σ is the twin wall formation energy, k is the proportionality factor, c is the elastic constant, *t* is the unit cell tetragonality (*c*/*a*), *w* is the twin lamella width, and g is the grain size.

**S4.2. Kinetical Considerations of Ferroelastic Domain Formation**

While the thermodynamic equilibrium model of ferroelastic domains provides valuable insight into their dependence on tetragonality and grain size, experimental observations often deviate from these predictions. For example, ferroelastic domains are frequently observed in free-standing grains isolated on carbon films, even in the absence of neighboring crystallites (Figure S7). In such cases, a single-domain configuration would be expected, as no external clamping is present to generate mechanical stress during the phase transition.

One possible thermodynamic explanation is that a surface layer remains in the paraelastic phase during the transition, exerting epitaxial stress on the underlying crystal. Alternatively, surface tension may induce localized stress. These effects imply that even free-standing grains can adopt multi-domain configurations, with the equilibrium state still influenced by factors such as grain size (or sample thickness) and tetragonality (i.e., the order parameter). However, since surface-induced effects diminish with increasing crystal size (scaling with 1/g), they cannot account for the presence of domain structures in large single crystals, such as MAPbI_3_ single crystals exceeding hundreds of microns in size.^[6]^

We therefore propose a kinetic origin for the formation of domain structures in free-standing ferroelastic crystals. This concept is analogous to the formation of polycrystalline microstructures, which often persist despite the thermodynamic instability of grain boundaries. Although the true equilibrium configuration of a free-standing ferroelastic crystal is a single-domain state, kinetic barriers may hinder the system from reaching this global minimum.

This kinetic behavior can be understood through the nucleation and growth process during the cubic-to-tetragonal phase transition. As the cubic unit cell (a = b = c) transforms into a tetragonal structure (a = b ≠ c), three distinct c-axis orientations become energetically favorable (Figure S8a). Tetragonal domains nucleate independently within regions of the parent cubic grain and grow with these different orientations. Domains with the same c-axis orientation coalesce, while those with different orientations form domain boundaries. When such mismatched domains meet along a pseudo-symmetry {112}ₜ plane, the interface can stabilize as a twin wall, resulting in the formation of {112}ₜ twin structures, as illustrated in Figure S8a-S8d.

This model accounts for the presence of domain structures even in the absence of external mechanical constraints. Nevertheless, the resulting configurations are metastable and not in thermodynamic equilibrium, and therefore may not exhibit a clear correlation with grain size or tetragonality.

**S4.3. The Evolution of Cross-Lamella Ferroelastic Domain Structures**

The coalescence of tetragonal domains can involve not only the merger of two domains but also the intersection of three or more, resulting in cross-lamellar configurations where twin boundaries intersect at approximately 90°. Diffraction analysis confirms that these orthogonal boundaries still belong to the {112}_t_ twin family (Figure S3). However, such intersecting configurations introduce angular misfit boundaries that are geometrically unstable due to intrinsic crystallographic constraints, as detailed in Note S5. Consequently, parallel {112}ₜ twin walls aligned in a single orientation are energetically more favorable than multi-oriented crossing configurations, consistent with the dominance of simple lamellar structures in purely ferroelastic systems.

Despite this, our experimental results reveal an increasing occurrence of cross-type domain structures with grain size. As shown in Figure S6, the fraction of twinned grains exhibiting cross-lamella configurations increases in larger MAPbI_3_ grains. This trend can be explained by considering the kinetic barrier associated with reorienting independently nucleated lamellar clusters into a uniform direction. During cooling, simple lamellae may nucleate in different regions of a grain with distinct orientations. In smaller grains, these clusters can more readily reorient into a common direction, minimizing interfacial energy. In contrast, larger grains present a higher energy barrier for such realignment. As a result, misaligned lamellar clusters often persist, forming geometrically unfavorable crossing configurations. This process is schematically illustrated in Figure S8b. Therefore, the increased prevalence of cross-lamella domains in larger grains is attributed to kinetic limitations that prevent domain realignment during the phase transition.

**Note S5. Angular misfit boundaries in crossing-type lamella structures**

When three or more tetragonal domains nucleate independently with different orientations and intersect, cross-lamella structures can form. Diffraction analysis confirms that all boundaries in these configurations correspond to the {112}ₜ twin family (Figure S3). Therefore, examining the crystallographic orientation relationships among multiple domains is equivalent to analyzing the angular relationships between the corresponding {112}ₜ twin walls.

In the tetragonal phase, four equivalent {112}ₜ planes—denoted A1, A2, B1, and B2—can be identified (Figure S14). Based on these, a total of 11 geometrically distinct intersection configurations are possible, which reduce to four unique cases when considering crystallographic equivalency:

(1) Case 1: Intersections of A1–A2 or B1–B2, corresponding to a junction along the ⟨100⟩_c_ direction.
(2) Case 2: Intersections of A1–B1, A1–B2, A2–B1, or A2–B2, corresponding to a junction along the ⟨111⟩_c_ direction.
(3) Case 3: Triple-wall intersections (e.g., A1–A2–B1, A1–A2–B2, B1–B2–A1, B1–B2–A2), which converge at the point (1/2, 1/2, 1/2).
(4) Case 4: The four-wall intersection (A1–A2–B1–B2), which also converges at (1/2, 1/2, 1/2).

Here, Cases 3 and 4 are combinations of the two fundamental intersection types—Case 1 and Case 2—and therefore inherit the angular misfit characteristics from those simpler configurations. As such, angular misfit boundaries are described primarily for Cases 1 and 2.

For case 1, which intersects along the <100>_c_ vector, there is inevitably an angle misfit of 2θ_<100>_ or 4θ_<100>_ as shown in top panels of Figure S15. For case 2, which intersects along the <111>_c_ vector, there is an angle misfit of 2θ_<111>_ or 5θ_<111>_ as shown in bottom panels of Figure S15. These misfit angles, derived as a function of tetragonality (t), are given by:

$$\theta_{<100>}=2\tan^{-1} t- \frac{\pi}{2}$$

(S3)

$$\theta_{<111>}=\tan^{-1} (\sqrt{1+2t^{2}})-\frac{\pi}{3}$$

(S4)

These angular misfit boundaries are inherently geometrically constrained and energetically unfavorable. Consequently, cross-lamella domain structures—particularly those involving triple or quadruple junctions—are intrinsically less stable than simple lamellar configurations, explaining their relative rarity and metastability in ferroelastic systems.

**Note S6. Measurement of Ferroelastic Twinning Probability as a Function of Grain Size.**

We examined the proportion of ferroelastic lamella-twinned grains in MA_1-x_Gua_x_PbI_3_ (for x = 0, 0.05, 0.1, 0.15), categorized by grain size. As discussed in Note S3, identifying twinned grains involves some ambiguity due to the influence of grain orientation, particularly in highly tilted grains where twin contrast can be obscured by bending-related features. Despite this limitation, many grains exhibit unmistakable signatures of twinned domains.

A subset of the raw data used for this analysis is shown in Figure S18. For selected images, all visible grains were classified as either twinned or non-twinned, and their individual grain sizes were measured. Within each grain size interval, the fraction of twinned grains was determined. This analysis was performed across multiple grain size ranges, spanning from tens of nanometers to a few micrometers. Although the actual twinning probability is likely higher than the measured fraction—due to limitations described in Note S3—this approach provides a reasonable approximation of the size-dependent twinning probability.

**Note S7. Low-dose atomic-resolution scanning transmission electron microscopy**

Atomic-resolution STEM of metal halide perovskites is constrained by their extreme beam sensitivity. Because the electron dose scales with the inverse square of the field of view, atomic-resolution imaging typically accumulates several thousand e⁻ Å⁻^2^, whereas the perovskite lattice already degrades at only tens of e⁻ Å⁻^2^ (Note S1). A single high-magnification scan can therefore destroy the structure, precluding the repeated scanning normally used for zone-axis alignment and lens fine-tuning. Reliable atomic imaging thus requires (i) an extremely low probe dose and (ii) a single-scan protocol that images random regions without prior area or zone-axis selection.^[7]^

Using such ultralow-dose conditions, we imaged the atomic structure of MA_1-x_Gua_x_PbI_3_. Figure S20 shows a {111}_c_ nanodomain of MA_0.8_Gua_0.2_PbI_3_ over three consecutive scans: the lattice and domain structures are clearly resolved in the first scan but largely destroyed by the second, confirming that these conditions yield reliable structural information from a single scan.

The reduced dose lowers the collected-electron count and thus the signal-to-noise ratio (SNR), which is especially limiting for HAADF-STEM owing to its low collection efficiency. Various approaches can mitigate this (e.g., LAADF-STEM,^[7]^ multislice electron ptychography,^[8,9]^ and machine-learning denoising); here we applied Bragg filtering to enhance feature visibility. Figures S21a-S21d show raw images with FFTs of MA_0.8_Gua_0.2_PbI_3_ and Figures S21i–S21p the corresponding Bragg-filtered images with atomic models (Figures S21q–S21t). Although Bragg filtering can suppress or distort information—making it unsuitable for precise atomic-position or strain measurements—it still provides reliable defect-structure distributions at improved SNR.

**Note S8. 4D-STEM NBD data-processing methodology**

From the low-dose 4D-STEM nanobeam diffraction (NBD) dataset I(R, Q) (R, real-space scan position; Q, diffraction-space coordinate), grains were segmented and defect-bearing grains were identified through the procedure below (the full workflow is summarized in Figure S24).

1) Rough NMF. For each scan position, the direct (transmitted) beam was masked and the diffraction pattern was binarized, so that only the peak positions — rather than the diffracted intensities — were used as features. Non-negative matrix factorization (NMF) was then applied to this set of binarized diffraction patterns to obtain an initial decomposition, in which each component consists of a real-space loading map (hereafter "feature") and a corresponding characteristic diffraction pattern. Removing the intensity information ensures that the NMF components group pixels according to crystallographic orientation (i.e., the set of excited reflections).

2) Grain separation. Each NMF feature map (in real space) was thresholded to extract well-defined connected regions (islands). Because two spatially separated grains can share a similar orientation and thus be assigned to a single NMF component, any component containing two or more disconnected islands was split into separate grains.

3) Grain merging. Conversely, a single grain can be split across multiple NMF components when an orientation mistilt changes which reflections are strongly excited, even though the grain is crystallographically continuous. To correct for this, spatially adjacent grain pairs were examined in real space, and two grains were merged into one whenever the similarity between their binarized diffraction patterns exceeded a set threshold. This separation-and-merging step yielded a cleaned grain label map, in which each grain corresponds to a single crystallographically coherent region. The area of each grain was converted from pixels to nm² using the calibrated pixel size (7.305–10.43 nm px⁻¹, magnification-dependent).

4) Defect-grain detection**.** Grains containing {111}_c_-type planar defects were identified from their representative NBD patterns. Because full crystallographic orientation indexing for every grain is impractical across the many grain orientations sampled, we used a conservative operational criterion based on the characteristic perovskite {111}_c_-related diffraction signature, rather than on streaking alone. Candidate grains were first screened for diffraction streaking. A grain was then labeled as defect-bearing only when a diffraction streak was collinear with a first-order diffraction peak corresponding to the perovskite {111}_c_ interplanar spacing (d ≈ 3.66 Å); grains showing no streaking, or streaking not assignable to a first-order perovskite {111}c reflection, were labeled as defect-free.

This first-order-peak-plus-collinear-streak criterion intrinsically excludes secondary-phase contributions. A δ-phase polytype could in principle produce a reflection near 3.66 Å through two routes, but neither satisfies the criterion. (i) Off-*c*-axis reflections: the δ-phase polytypes (2H, 4H, 6H, 8H) can exhibit interplanar spacings near 3.66 Å that do not lie along the hexagonal *c*-axis (for example, 8H {107} = 3.65 Å, 6H {202} = 3.62 Å). Since streaking from face-sharing stacking develops only along the hexagonal c-axis—crystallographically equivalent to the {111}_c_ stacking direction of the cubic (3C) perovskite—these off-*c*-axis reflections cannot appear as a streak collinear with the 3.66 Å peak. (ii) *c*-axis reflections: the *c*-axis reflections of the *δ* polytypes do not fall at 3.66 Å (2H {002} = 3.95 Å, 4H {004} = 3.80 Å, 6H {006} = 3.74 Å, 8H {008} = 3.63 Å). The values nearest 3.66 Å, 6H {006} = 3.74 Å and 8H {008} = 3.63 Å, are not first-order reflections but higher-order {00l} reflections, each necessarily accompanied by its lower-order counterparts ({002}/{004} for 6H, and {002}/{004}/{006} for 8H) at smaller angles (larger d). Because our criterion indexes a grain as {111}_c_ only when 3.66 Å appears as the first-order reflection along the streaking direction, such higher-order *δ*-phase cases are excluded by construction. More generally, the first-order *c*-axis reflection of any *δ* polytype corresponds to its full stacking period and therefore lies at a substantially larger d-spacing than 3.66 Å, so no δ polytype places a first-order c-axis reflection at this spacing. Consequently, a pattern exhibiting a d ≈ 3.66 Å first-order peak together with a collinear streak along that direction can arise only from {111}c planar defects in the 3C perovskite structure. δ-phase polytypes therefore fail this criterion, which exhibit a distinct set of reflections; both were excluded from the {111}c defect-bearing count.

Diffraction patterns unambiguously assignable to long-range-ordered δ-phase polytypes were identified separately, as summarized in Figure S31. The representative per-grain NBD patterns are provided in Figures S26 and S29, and the {111}c-oriented streaks used for the classification across all ROIs are summarized in Figures S27 and S30. All raw data, processing code, and analysis outputs supporting this classification are available in a public GitHub repository, provided in Data Availability Statement.

**Note S9. Area-dependent detection bias in the 4D-STEM defect-grain statistics**

Defect-bearing grains have a slightly larger median area than defect-free grains (Figure 5c). We attribute this to area-sampling bias rather than to preferential defect formation in larger grains: because the planar defects traverse the grain and are flagged whenever a single diffraction streak is present, a larger grain is more likely to contain at least one defect simply because it comprises more lattice planes, even at a fixed, size-independent defect line density.

To quantify this, we model defect detection as a random per-plane process and fit the resulting incidence probability, P(A) = 1 − exp(−λA^β^), to the per-grain binary data (defect-bearing vs. defect-free) by complementary log–log Bernoulli regression. For planar defects that span the grain, the defect count scales with the number of {111} planes and hence with the grain's linear dimension (∝ A^1/2^); a constant line density therefore predicts β = 1/2, which we adopt as the null hypothesis of no intrinsic size dependence (β > 1/2 would indicate a genuine preference for larger grains).

The fits give β = 0.41 for Gua20 (n = 834, 16 defect-bearing) and β = 0.27 for Gua25 (n = 573, 58 defect-bearing). Both are statistically consistent with the β = 1/2 null (likelihood-ratio test p = 0.70 and 0.085) and neither point estimate exceeds it, so the observed size difference is largely explained by the geometry of grain-spanning planar defects at constant line density, with no evidence of preferential formation in larger grains. This supports an origin in stacking-sequence errors introduced during grain growth, without a critical grain-size dependence, in agreement with bright-field TEM observations on the same specimens (Figure S23).

**Figure S1. Electron beam-induced degradation of perovskite lattice structures** a) Selected Area Diffraction Pattern (SADP) of the film showing the ring pattern by polycrystalline MAPbI_3_. The accumulated electron dose was noted at the top right (yellow). Dose rate of 0.08 e⁻ Å⁻^2^ s⁻^1^ was used. b) Radial intensity distribution of the ring pattern from (a). The peak near spatial frequency of 1.6 nm^-1^ corresponds to the {110}_t_ lattice plane of MAPbI_3_.

**Figure S2. Electron Beam Damage Experiments for Intragrain Planar Defect Structure Observation**. a) BF TEM images of MAPbI_3_ film synthesized under the SA30 condition, with grains exhibiting {112}_t_ ferroelastic twin domain structures. b) BF TEM images of MA_0.8_Gua_0.2_PbI_3_ film synthesized under the IA30 condition, showing {111}_c_ planar defects. The accumulated electron dose is indicated in the top-right corner of each image.

**Figure S3. Selected Area Electron Diffraction Patterns (SADPs) of {112}ₜ Ferroelastic Twins in MAPbI_3_ Films**. a) SADP of a simple lamellar twin structure showing peak splitting along the (1-12) direction but not along the (112) direction. b) Kinematical diffraction simulation result of {112}t-twinned structures corresponding to (a). c) SADP of a cross-lamellar twin structure showing peak splitting along both the (1-12) and (112) directions. d) Kinematically simulated diffraction pattern corresponding to (c). Electron diffraction simulations were performed using the SingleCrystal software.

**Figure S4.** **Grain Size Analysis under Different Annealing Conditions.** a) Schematic illustration of inert annealing (IA) and solvent annealing (SA). Anhydrous N,N-dimethylformamide and dimethyl sulfoxide mixed solvent (9:1 v/v) vapor was used for SA. b) Top-view scanning electron microscopy (SEM) images of MAPbI_3_ films prepared under IA30, SA10, and SA30 conditions. Scale bar: 1 μm. c) Grain size distributions of MAPbI_3_ films under the three annealing conditions, measured from the SEM images.

**Figure S5. X-ray Diffraction (XRD) Analysis of MA_1-x_Gua_x_PbI_3_ Films under Different Annealing Conditions.** a,b) XRD patterns of films annealed under IA30 (a) and SA30 (b) conditions. Films with guanidinium (Gua) concentrations of 0%, 5%, 10%, 15%, 20%, 25%, 40%, and 100% were analyzed. For the MAPbI_3_ (Gua 0%) films, no significant variation in peak positions was observed between the two annealing conditions. Enlarged views of the perovskite {110}ₜ peaks near 14° (right panels) show a gradual lattice expansion, indicating A-site substitution by Gua. The Gua40 and Gua100 samples are included only as over-doped reference samples for assigning the Gua-rich/δ-related diffraction features and are excluded from the quantitative defect, device, and EQE comparisons in the main text.

**Figure S6. Grain size dependence of the evolution of crossing-type lamellae.** Plot showing the ratio of cross-lamella grains to all twinned grains as a function of grain size, extracted from BF TEM images of MAPbI_3_ films annealed under IA30, SA10, and SA30 conditions.

**Figure S7. Ferroelastic twin stripes in isolated grains.** Bright-field TEM image of a MAPbI_3_ film. The magnified region on the right highlights domain structures observed in grains that are not laterally clamped by surrounding grains.

**Figure S8. Schematic illustration of the ferroelastic twin domain formation mechanism.** a) Three possible orientations of the tetragonal unit cell that emerge from the cubic phase during the cooling-induced phase transition. b) Schematic of the joining process between two simple lamella clusters originating from different regions within a single grain during the phase transition. Two cases with different grain sizes are illustrated. c) Evolution of tetragonal domain structures within grains of three different sizes during the cooling process. d) Equilibrium domain configurations with and without external constraints. e) Energetic competition between phase transition-induced strain fields and twin wall formation energies.

**Figure S9. Schematic definition of normal and parallel lengths for ferroelastic domain analysis.** The normal length (L_N_) is defined as the grain length perpendicular to the domain wall, used for counting domain wall intersections. The parallel length (L_P_) refers to the grain length parallel to the domain wall.

**Figure S10. Measurement of lamella width and grain size from BF TEM images of MAPbI_3_ films.** Grains exhibiting well-defined ferroelastic domains suitable for lamella width analysis were labeled with index numbers (#1–#106). a–x) BF TEM images of films annealed under IA30 (t–x), SA10 (a, b), and SA30 (c–s) conditions. The corresponding lamella width values are listed in Table S1.

**Figure S11. Log–log scatter plot of lamella width versus grain size.** Lamella widths measured from BF TEM images (Table S1) are plotted as a function of grain size on a logarithmic scale. Data points are color-coded according to annealing condition: IA30 (green), SA10 (yellow), and SA30 (red).

**Figure S12. Visibility of ferroelastic domain structures in MAPbI_3_ observed by BF TEM.**
Atomic models of MAPbI_3_ perovskite with octahedral frameworks illustrate {112}t twin walls. Corresponding BF TEM images show ferroelastic domain structures with varying contrast features.

**Figure S13. Tilt-series BF TEM images of ferroelastic twin domain structures.** Images were acquired at tilt angles of 0°, 2°, and 4° along a single axis. A composite color image is shown on the right, where red, green, and blue represent 0°, 2°, and 4°, respectively.

**Figure S14. Possible crystallographic orientational configurations of {112}ₜ twin walls.** Tetragonal unit cell with lattice parameters a, a, and c is shown on the left. Possible orientation configurations are classified into four groups from case 1 to case 4, depending on the intersections between two or more {112}_t_ twin planes.

**Figure S15. Schematic of angular misfit at crossing-type lamella boundaries.** Case 1 and Case 2 in Figure S14 lead to formation of *θ*_<010>_ and *θ*_<111>_ angular misfit boundaries, respectively, as detailed in Note S5.

**Figure S16. Tetragonality and unit-cell parameters of MA_1-x_Gua_x_PbI_3_ extracted from XRD.** XRD patterns of MA_1-x_Gua_x_PbI_3_ films annealed under IA30 (inert annealing, 30 min) and SA30 (solvent annealing, 30 min) conditions. In both cases, the {202}_t_ peak—structurally equivalent to the {111}_c_ peak—and the {110}_t_ peak were used to determine the unit-cell parameters. The tetragonal lattice parameter a was obtained from the {110}*_t_* reflection through d(110) = *a*/√2 (i.e., *a* = √2·*d*(110)), and c from the {202}t reflection using this a; each d-spacing follows from Bragg's law, λ = 2d sinθ (Cu Kα1, λ = 1.5406 Å). Tetragonality is expressed on a pseudo-cubic scale and computed from the tetragonal cell as t = c/(√2·a) (equivalently, the pseudo-cubic c/a). (a, d) {202}t (red) and {110}t (green) peaks with single-Gaussian-plus-baseline fits for IA30 (a) and SA30 (d). (b, e) Extracted lattice parameters *a* (tetragonal) and *c* for IA30 (b) and SA30 (e). (c, f) Corresponding tetragonality *t* for IA30 (c) and SA30 (f). The uncertainty in the extracted parameters was assessed against two sources. First, to check for systematic instrumental error (e.g., sample displacement or zero-point offset, which shifts all peaks in common), we compared the measured MAPbI_3_{110}_t_ position in our films (2θ = 14.16°, i.e., *d*(110) = 6.25 Å, giving *a* = √2·*d*(110) = 8.838 Å) with reported reference values for tetragonal MAPbI_3_. The agreement is within 0.02° in 2θ (a= 8.836 Å, 2θ(110) = 14.16°^[10]^ and *a* = 8.849 Å, 2θ(110) = 14.14°^[11]^) confirming that no significant systematic shift was introduced. Second, peak positions were obtained by Gaussian fitting; the standard error of each fitted peak center was propagated through Bragg's law and the lattice-parameter relations to obtain the uncertainties. Because *t* is a ratio of *c* and *a*, any residual common shift in absolute peak position largely cancels in this fractional form. Numerical *t* values with their propagated uncertainties are annotated in panels (c) and (f).

**Figure S17. Grain sizes as a function of Gua concentration in MA1-xGuaxPbI3 films.** a) Scanning Electron Microscopy Top-view Images of MA_1-x_Gua_x_PbI_3_ Films under IA30 with Gua concentrations of 0, 5, 10, 15, 20, and 25 %. Scale bar: 400 nm. b) Plot of the measured grain size distributions.

**Figure S18. BF TEM images of MA_1-x_Gua_x_PbI_3_ films under IA30 and SA30****.** a) BF TEM images of films with 0%, 5%, 10%, and 15% Gua concentrations under IA30 (top) and SA30 (bottom). b) BF TEM images of two regions of interest (ROI 1 and ROI 2) for Gua 10 (top) and Gua 15 (bottom) under SA30, with lamella twin-bearing grains outlined by dashed lines.

**(1-11)**

**(1-11)**

**(1-10)**

**(1-10)**

**(1-10)**

**(1-10)**

**Figure S19. Selected-area diffraction patterns of {111}_c_ planar defects in MA_0.8_Gua_0.2_PbI_3_.** TEM-SADP (left) is provided with corresponding simulated diffraction patterns showing {111}_c_ twin structures. Electron diffraction simulation (kinematical) was conducted using Single Crystal code.

**Figure S20. Electron beam tolerance of MA_1-x_Gua_x_PbI_3_ for atomic-resolution STEM.** STEM-high angle annular dark field (HAADF) images of MA_0.8_Gua_0.2_PbI_3_ film with fast Fourier transform (FFT) images as inset. Three consecutive scans were conducted on the same region. An enlarged view of the region of interest marked by the orange rectangle is provided below each image.

**Figure S21. Atomic-resolution HAADF-STEM images of MA_0.8_Gua_0.2_PbI_3_ perovskite films.** a–d) Raw images along the perovskite <110> a), <100> b), <100> with a twin boundary c), and PbI_2_ <100> zone axes d). e–h) Corresponding FFT patterns. i–l) Bragg-filtered images of a–d). m–p) Magnified views of the insets in i–l). q–t) Atomic models of the perovskite q–s) and PbI_2_ t).

**Figure S22. DFT-calculated formation energy of {111}_c_ twins as a function of tetragonality.** a) The fully-relaxed unit cell parameters of the cubic and tetragonal phase are provided. b) The atomic model used to calculate {111}_c_ twin formation energy. c) The map of total system energy of MAPbI_3_ unit cell with two variables: tetragonality and unit cell volume. d) The twin wall formation energy as a function of tetragonality. The tetragonality was varied with fixed unit cell parameters, along with the dashed line in (c).

**Figure S23. Grain size effects on the formation of {111}_c_ planar defects.** BF TEM images of MA_0.75_Gua_0.25_PbI_3_ films. Reddish circles highlight the features of {111}_c_ planar defects showing the formation of these defects is weakly correlated with grain size.

**Figure S24. 4D-STEM NBD workflow for grain segmentation and defect-grain classification.** Data-processing pipeline applied to the 4D-STEM NBD dataset, detailed in Note S8.

**Figure S25. Grain-by-grain 4D-STEM NBD defect quantification for Gua20 (MA_0.8_Gua_0.2_PbI_3_)**. Grain-label maps of the four analyzed regions of interest (ROIs), overlaid on the corresponding virtual bright-field images, with each grain colored defect-bearing (red) or defect-free (gray) according to the classification in Note S8. Scale bars: 100 nm.

**Figure S26. Representative per-grain NBD patterns (ROI1) for Gua20 (MA_0.8_Gua_0.2_PbI_3_).** NBD pattern of every segmented grain. Patterns exhibiting diffraction streaking along {111}_c_ directions are outlined in red and classified defect-bearing; the remaining grains are classified defect-free.

**Figure S27. {111}_c_-oriented streaks in the grain-by-grain diffraction patterns of Gua20.** Diffraction patterns containing {111}_c_-oriented streaks among the grain-by-grain diffraction patterns of Gua20, from ROI1 to ROI4 (arranged from top to bottom, corresponding to data collected from ROI1, 2, 3, and 4, respectively). The red circles indicate the peaks corresponding to the {111} interplanar spacing of d = 3.66 Å. Scale bar, 2 nm^-1^

**Figure S28. Grain-by-grain 4D-STEM NBD defect quantification for Gua25 (MA_0.75_Gua_0.25_PbI_3_).** Grain-label maps of the four analyzed regions of interest (ROIs), overlaid on the corresponding virtual bright-field images, with each grain colored defect-bearing (red) or defect-free (gray) according to the classification in Note S8. Scale bars: 100 nm.

**Figure S29. Representative per-grain NBD patterns (ROI1) for Gua25 (MA_0.75_Gua_0.25_PbI_3_).** NBD pattern of every segmented grain. Patterns exhibiting diffraction streaking along {111}_c_ directions are outlined in red and classified defect-bearing; the remaining grains are classified defect-free.

**Figure S30. {111}_c_-oriented streaks in the grain-by-grain diffraction patterns of Gua25.** Diffraction patterns containing {111}-oriented streaks among the grain-by-grain diffraction patterns of Gua25, from ROI1 to ROI4 (arranged from top to bottom, corresponding to data collected from ROI1, 2, 3, and 4, respectively). The red circles indicate the peaks corresponding to the {111} interplanar spacing of d = 3.66 Å. Scale bar, 2 nm^-1^

**Figure S31. Identification and distribution of the δ phase.** (a) Grain segmentation map obtained from Gua20, corresponding to ROI1. (b) Virtual bright-field (v-BF) image of the same dataset overlaid with the {111}_c_-defect grains and the δ-phase grains; in particular, the blue regions denote δ-phase domains indexed as the 8H polytype, corresponding to grain indices 62 and 201. (c) Diffraction patterns obtained from the real-space regions corresponding to grain indices 62 and 201. (d) Interpolated diffraction-pattern data of grain indices 62 and 201 (interpolation applied to improve the accuracy of peak detection), together with the simulated diffraction pattern of the 8H phase along the <110> zone axis and the corresponding atomic model of the 8H phase viewed along the same orientation. This pattern is unique to the 8H polytype and cannot arise from the other δ-phase polytypes (2H, 4H, and 6H).

**Figure S32. Intragrain strain distribution in Gua25 from 4D-STEM NBD.** (Note: these data were acquired with a long camera length of 1.8 m and a magnification of 100 kX to improve the accuracy of the strain maps.) (a) v-BF image of Region 1 (top left) and the diffraction pattern from the grain interior (boxed region); below is the strain map of the perovskite 001 zone-axis region extracted via NMF. (b) Histograms of the frequency of each strain component obtained from the Region 1 strain map. (c) v-BF image of Region 2 (top left) and the diffraction pattern from the grain interior (boxed region); below is the strain map of the perovskite 001 zone-axis region extracted via NMF. (d) Histograms of the frequency of each strain component obtained from the Region 2 strain map. (e) Mapping of only the regions corresponding to strain > +1% (red) and < −1% (green) extracted from the Region 2 strain map (leftmost), the corresponding average diffraction patterns (red and green), and, on the far right, an overlay of the two diffraction patterns (the inset shows the splitting of the peaks).

**Figure S33. Time-resolved PL spectra of the perovskite absorber layers with varied Gua content.**

**Figure S34. Photovoltaic performance of single-junction devices with varied Gua content.**

**Figure S35. EQE spectra of optimized perovskite solar cells with different Gua ratios**

**Figure S36. Optical bandgap estimation from Tauc plots of Gua-containing films**

**Figure S37. EIS spectra of MA_1-x_Gua_x_PbI_3_ solar cells (Gua 0, 15, and 40%).**

**Table S1. Statistics of lamella domain wall intersections versus grain size in MAPbI_3_**

| **Index** | **Normal Length (μm)** | **Parallel Length (μm)** | **# of intersection** |
| --- | --- | --- | --- |
| 1 | 0.393 | 0.430 | 4 |
| 2 | 0.485 | 0.473 | 7 |
| 3 | 0.232 | 0.300 | 5 |
| 4 | 0.535 | 0.484 | 6 |
| 5 | 0.371 | 0.482 | 5 |
| 6 | 0.360 | 0.372 | 5 |
| 7 | 0.445 | 0.441 | 5 |
| 8 | 0.439 | 0.426 | 7 |
| 9 | 0.397 | 0.332 | 3 |
| 10 | 0.417 | 0.449 | 4 |
| 11 | 0.643 | 0.577 | 6 |
| 12 | 0.299 | 0.312 | 2 |
| 13 | 0.320 | 0.220 | 2 |
| 14 | 0.367 | 0.395 | 4 |
| 15 | 0.250 | 0.269 | 5 |
| 16 | 0.245 | 0.238 | 5 |
| 17 | 0.354 | 0.296 | 6 |
| 18 | 0.311 | 0.339 | 3 |
| 19 | 0.294 | 0.363 | 4 |
| 20 | 0.379 | 0.247 | 4 |
| 21 | 0.465 | 0.418 | 5 |
| 22 | 0.378 | 0.400 | 5 |
| 23 | 0.345 | 0.342 | 6 |
| 24 | 0.297 | 0.335 | 5 |
| 25 | 0.379 | 0.424 | 3 |
| 26 | 0.518 | 0.390 | 4 |
| 27 | 0.213 | 0.254 | 3 |
| 28 | 0.399 | 0.340 | 4 |
| 29 | 0.334 | 0.302 | 5 |
| 30 | 0.369 | 0.372 | 4 |
| 31 | 0.28 | 0.273 | 3 |
| 32 | 0.229 | 0.264 | 2 |
| 33 | 0.499 | 0.453 | 3 |
| 34 | 0.398 | 0.357 | 4 |
| 35 | 0.286 | 0.250 | 3 |
| 36 | 0.302 | 0.355 | 3 |
| 37 | 0.301 | 0.303 | 3 |
| 38 | 0.320 | 0.340 | 4 |
| 39 | 0.422 | 0.325 | 5 |
| 40 | 0.44 | 0.527 | 7 |
| 41 | 0.419 | 0.418 | 3 |
| 42 | 0.315 | 0.362 | 4 |
| 43 | 0.365 | 0.324 | 4 |
| 44 | 0.410 | 0.490 | 2 |
| 45 | 0.230 | 0.210 | 3 |
| 46 | 0.828 | 0.810 | 8 |
| 47 | 1.109 | 1.138 | 15 |
| 48 | 0.884 | 0.984 | 9 |
| 49 | 0.598 | 0.427 | 4 |
| 50 | 0.744 | 0.770 | 8 |
| 51 | 0.578 | 0.776 | 6 |
| 52 | 0.400 | 0.978 | 5 |
| 53 | 0.972 | 0.997 | 8 |

| **Index** | **Normal Length (μm)** | **Parallel Length (μm)** | **# of intersection** |
| --- | --- | --- | --- |
| 54 | 1.040 | 1.021 | 12 |
| 55 | 0.811 | 0.750 | 6 |
| 56 | 0.972 | 0.142 | 11 |
| 57 | – | – | – |
| 58 | 0.714 | 0.517 | 6 |
| 59 | – | – | – |
| 60 | – | – | – |
| 61 | 1.740 | 1.740 | 16 |
| 62 | 0.888 | 1.150 | 9 |
| 63 | 1.566 | 1.663 | 18 |
| 64 | 0.653 | 0.460 | 8 |
| 65 | 1.493 | 1.372 | 17 |
| 66 | 0.713 | 0.683 | 3 |
| 67 | 0.574 | 0.491 | 7 |
| 68 | 1.299 | 1.178 | 8 |
| 69 | – | – | – |
| 70 | 0.663 | 0.678 | 6 |
| 71 | 1.097 | 1.079 | 14 |
| 72 | 0.783 | 0.616 | 11 |
| 73 | 1.015 | 0.999 | 10 |
| 74 | 1.355 | 1.160 | 9 |
| 75 | 0.680 | 1.052 | 4 |
| 76 | 0.765 | 0.509 | 8 |
| 77 | 1.406 | 1.406 | 14 |
| 78 | 1.480 | 1.416 | 12 |
| 79 | 2.904 | 1.710 | 24 |
| 80 | 2.300 | 1.700 | 16 |
| 81 | 2.126 | 2.460 | 20 |
| 82 | 1.469 | 1.225 | 9 |
| 83 | 1.050 | 1.039 | 10 |
| 84 | 1.247 | 1.014 | 11 |
| 85 | 1.653 | 1.510 | 11 |
| 86 | 1.120 | 1.340 | 8 |
| 87 | 1.140 | 2.320 | 11 |
| 88 | 1.610 | 2.070 | 10 |
| 89 | 1.195 | 1.090 | 13 |
| 90 | 1.670 | 2.020 | 10 |
| 91 | 1.450 | 1.970 | 12 |
| 92 | 2.130 | 2.050 | 17 |
| 93 | 1.859 | 1.372 | 14 |
| 94 | 1.655 | 1.365 | 13 |
| 95 | 2.034 | 3.100 | 20 |
| 96 | 1.410 | 3.010 | 8 |
| 97 | 0.834 | 0.875 | 12 |
| 98 | 1.125 | 1.130 | 13 |
| 99 | 0.347 | 0.288 | 6 |
| 100 | 0.247 | 0.224 | 4 |
| 101 | 0.166 | 0.200 | 3 |
| 102 | 0.227 | 0.313 | 4 |
| 103 | 0.180 | 0.162 | 4 |
| 104 | 0.184 | 0.202 | 3 |
| 105 | 0.231 | 0.254 | 4 |
| 106 | 0.321 | 0.281 | 6 |

*Note: Domain structures that had not reached equilibrium were excluded from the count and are marked with “–”*

**Table S2.** Urbach energy (E_U_) of MA_1-x_Gua_x_PbI_3_ perovskite films.

|  | *E*_U_ (meV) |
| --- | --- |
| Gua 0 | 17.31 |
| Gua 5 | 17.13 |
| Gua 10 | 16.93 |
| Gua 15 | 16.60 |
| Gua 20 | 21.65 |
| Gua 25 | 24.34 |

**Reference**

[1]	M. U. Rothmann, W. Li, Y. Zhu, A. Liu, Z. Ku, U. Bach, J. Etheridge, Y.-B. Cheng, *Advanced Materials* **2018**, *30*, 1800629.

[2]	M. U. Rothmann, W. Li, Y. Zhu, U. Bach, L. Spiccia, J. Etheridge, Y.-B. Cheng, *Nat Commun* **2017**, *8*, 14547.

[3]	A. L. Roitburd, *physica status solidi (a)* **1976**, *37*, 329.

[4]	G. Arlt, *J Mater Sci* **1990**, *25*, 2655.

[5]	I. A. Luk’yanchuk, A. Schilling, J. M. Gregg, G. Catalan, J. F. Scott, *Phys. Rev. B* **2009**, *79*, 144111.

[6]	I. M. Hermes, A. Best, L. Winkelmann, J. Mars, S. M. Vorpahl, M. Mezger, L. Collins, H.-J. Butt, D. S. Ginger, K. Koynov, S. A. L. Weber, *Energy Environ. Sci.* **2020**, *13*, 4168.

[7]	M. U. Rothmann, J. S. Kim, J. Borchert, K. B. Lohmann, C. M. O’Leary, A. A. Sheader, L. Clark, H. J. Snaith, M. B. Johnston, P. D. Nellist, L. M. Herz, *Science* **2020**, *370*, eabb5940.

[8]	G. Li, H. Zhang, Y. Han, *ACS Cent. Sci.* **2022**, *8*, 1579.

[9]	G. Li, M. Xu, W.-Q. Tang, Y. Liu, C. Chen, D. Zhang, L. Liu, S. Ning, H. Zhang, Z.-Y. Gu, Z. Lai, D. A. Muller, Y. Han, *Nat Commun* **2025**, *16*, 914.

[10]	M. T. Weller, O. J. Weber, P. F. Henry, A. M. D. Pumpo, T. C. Hansen, *Chem. Commun.* **2015**, *51*, 4180.

[11]	C. C. Stoumpos, C. D. Malliakas, M. G. Kanatzidis, *Inorg. Chem.* **2013**, *52*, 9019.
